# Supplementary material for: An RNA sponge directs the transition from feast to famine in Caulobacter crescentus
Source: Nat Commun. 2025 Oct 27;16:9478. doi: 10.1038/s41467-025-65274-1 (PMC12559287; doi:10.1038/s41467-025-65274-1)
Supplement: Supplementary file 1 — Supplementary Information [file 41467_2025_65274_MOESM1_ESM.pdf]

Supporting Information for

An RNA sponge directs the transition from feast to famine in *Caulobacter crescentus*

Laura N. Vogt, Manuel Velasco Gomariz, Malte Siemers, Kai Papenfort, Kathrin S. Fröhlich

corresponding author: Kathrin Sophie Fröhlich

Email: kathrin.froehlich@uni-jena.de

This PDF file includes:

Figures S1 to S10
Tables S1-S3
SI References

Other supporting materials for this manuscript include the following:

Supplementary Data 1-2

Supplementary Figure S1 - RIL-seq analysis in *C. crescentus*.

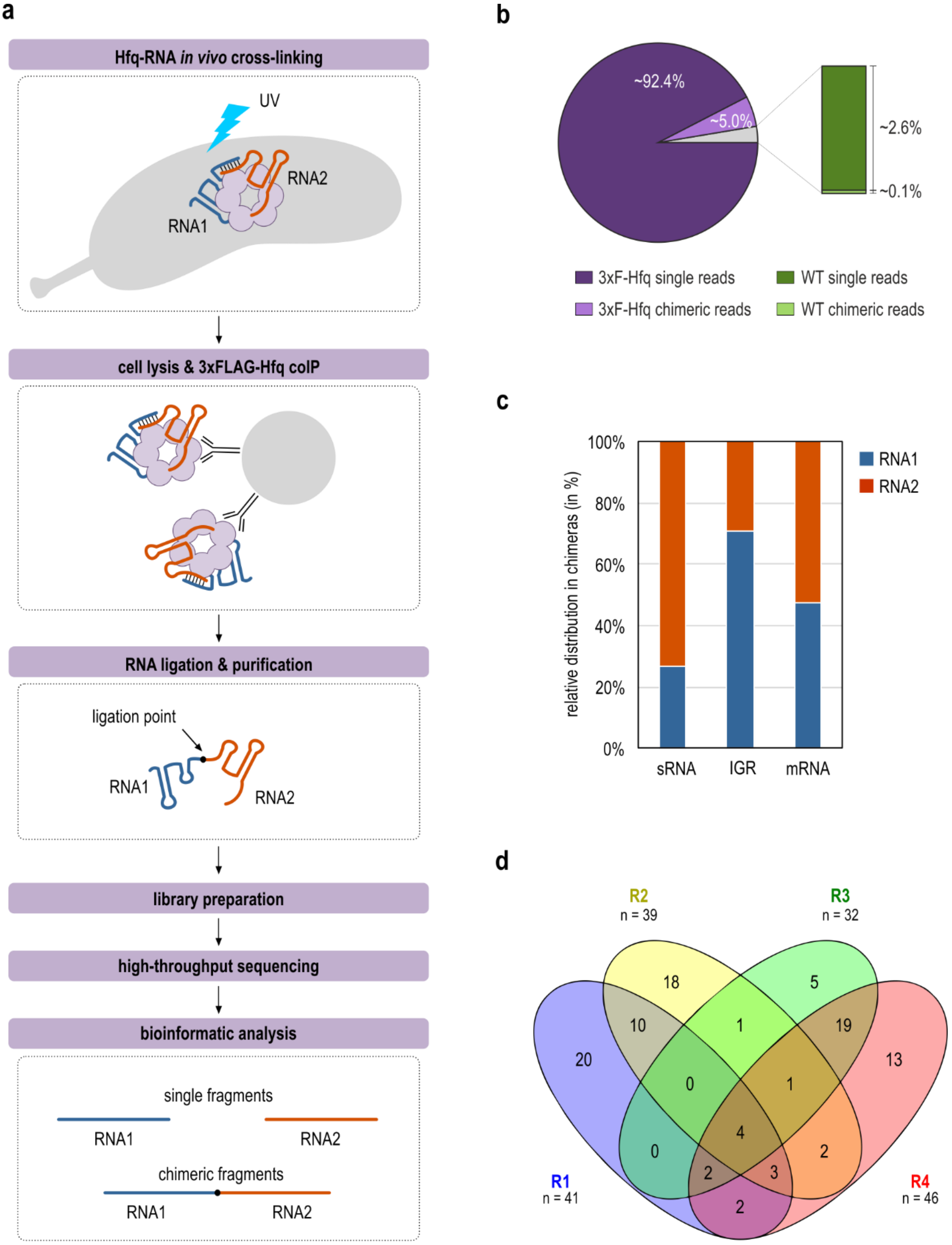

a Schematic representation of the RIL-seq workflow. *C. crescentus* WT and *hfq::3xFLAG* cells grown to OD_{660} of 1 in PYE were UV-crosslinked *in vivo*. After cell lysis, Hfq-bound RNAs were co-immunoprecipitated with 3xFLAG-Hfq using anti-FLAG antibodies conjugated to magnetic beads. Trimmed RNAs were ligated and purified. cDNA libraries were analysed by paired-end high-throughput sequencing. Reads were classified as single fragments with both reads mapping to one distinct genomic location or chimeric fragments with each read mapping to an independent site in the genome. Chimeric fragments represent potential interactions between transcripts. **b** Distribution of single and chimeric reads in RIL-seq samples of wild-type (WT) or *3xFLAG::hfq* (3xF-Hfq) cells. **c** Relative distribution of RNA classes in chimeric reads. **d**

Representation of the 100 most abundant high-confidence chimeras by combined reads in the individual 3xF-Hfq replicates (R1-R4). The number of unique chimeras of each replicate among the 100 most abundant is indicated (n). Source data for this figure are provided in the Source Data file**.**

Supplementary Figure S2 – RIL-seq chimera validation.

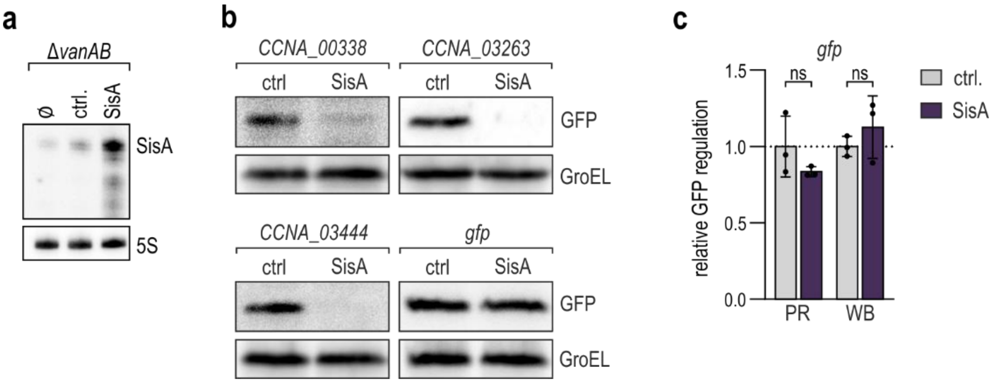

a Expression of SisA *C. crescentus* Δ*vanAB* cells carrying either no plasmid (Ø), empty control plasmid pBVMCS-6 (ctrl.) or pP_{van}-SisA. RNA was extracted from cells grown in PYE in the presence of vanillate to OD_{660} of 1.0 and analysed by Northern blot. 5S rRNA served as loading control. **b** Western blot analysis of *gfp* reporter fusions for *CCNA_00338*, *CCNA_03263*, *CCNA_03444* and the *gfp* control construct in combination with either an empty control vector (pBVMCS-6; ctrl.) or the expression plasmid pPvan-SisA. **c** Regulation of a *gfp* control construct by SisA. GFP expression was quantified either by fluorescence intensity measurements or by Western blot analysis of total protein samples as described in Fig. 1e. GFP levels were calculated as described in Fig. 1e as mean values ± SD of three biological replicates (n = 3). Statistical significance was assessed using unpaired two-sided Welch's t-tests (ns for $p > 0.05$). Source data for this figure are provided in the Source Data file.

Supplementary Figure S3 - Genomic location and conservation of *sisA-D*.

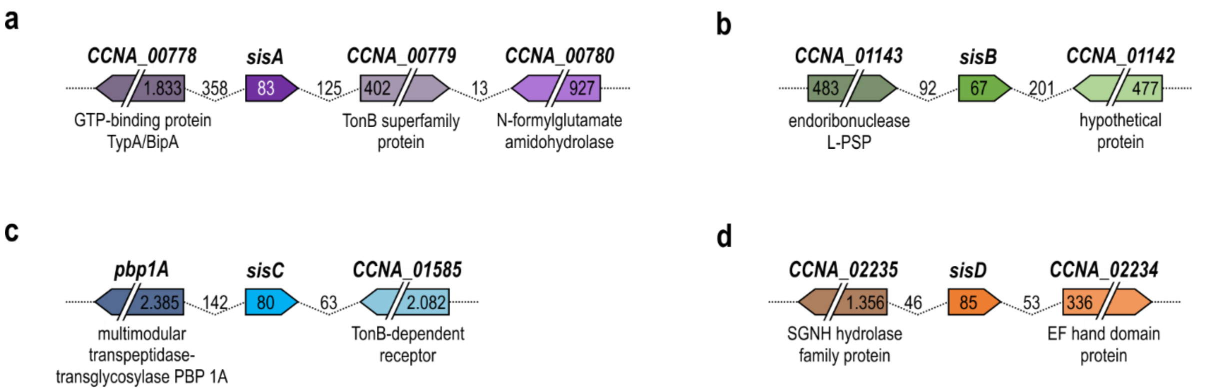

e

```
      -120                -100                      -80
       |                   |                         |
sisA   GAGCGTTGTTCTCCTTCCCCTTTAG-----GTAAAGGGTTTTTGCGAGGGTGAGCTTCAAAAAAA-
sisB   TCGAGGTCCAGGCCGCCAAGAAGAA--ATAGGCCTCGGCCGTCACCGGCCGGATTTCACCGTTACG
sisC   GCAGGCCCTCAATCGTCGAGCGCGCTCATATCACACGGCCGGGGCAAGCCA-AGCGCGCGGCTGA-
sisD   GTTCGCCAAGAGGCGTAAAGCGGCG--ATCGGAAGGGTTTGCTGCTGGGGTGGTGCTGCGACCGCT

       -60                 -40                  -20                  +1
       |                   |                    |                    |
sisA   TGACGTCGAACGTTTGTTTTCCGCTTGCCTAATTTTGCGCCGCACTCTAAAAACAATCCTG
sisB   CGAAATTATTCTCGTGACAAC--GTTGTC-AAGGCGAGGTCAAGTAGCTATATGGAACTCA
sisC   CGAAGAATTTTCAA-GGCGGC-TCTTGAACACCCGAAAAGTGAACATAGATCACCAAC--G
sisD   CGTCG-CGGCCACTAGGAATC-GCTTGCAACTGGACCTGTGTTAGCGCTATCAATCGCGTG
```

a-d Genomic context for *sisA-D* in *C. crescentus* with annotations for neighbouring genes. Gene sizes and distances to flanking genes are indicated in bp. **e** Alignment of the sequences upstream of *sisA-D* in *C. crescentus*. Colour indicates full (red), partial (blue) or no (black) conservation. A conserved TTG motif centred around position -35 is indicative of a recognition site for RpoD. The TSS (+1) of the sRNAs is underlined.

Supplementary Figure S4 - Expression of SisA-D family and CrfA in different media.

a

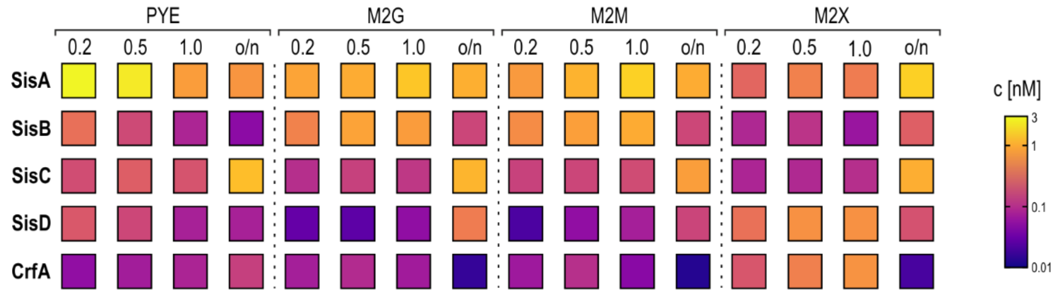

b

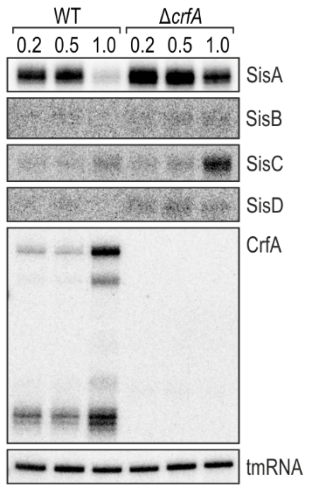

a Quantification of absolute SisA-D and CrfA levels over growth in different media. *C. crescentus* wild-type was grown in PYE, minimal medium supplemented with glucose (M2G), maltose (M2M) or xylose (M2X) as carbon source. Total RNA samples were collected over growth at OD_{660} of 0.2, 0.5, 1.0 and after over-night growth, respectively, analysed by Northern blot, shown in Fig. **2e** and quantified using dilution series of *in vitro* RNA of the respective sRNA. Colour gradient is displayed for log-transformed values. **b** Northern blot analysis of SisA-D and CrfA expression over growth in PYE in the presence (WT) or absence of CrfA (Δ*crfA*). *C. crescentus* wild-type or Δ*crfA* were grown in PYE. RNA extracted at OD_{660} of 0.2, 0.5 and 1.0, respectively, was analysed by Northern blot. tmRNA served as loading control. Source data are provided as a Source Data file.

Supplementary Figure S5 – Conservation of the SisA-D sRNA family

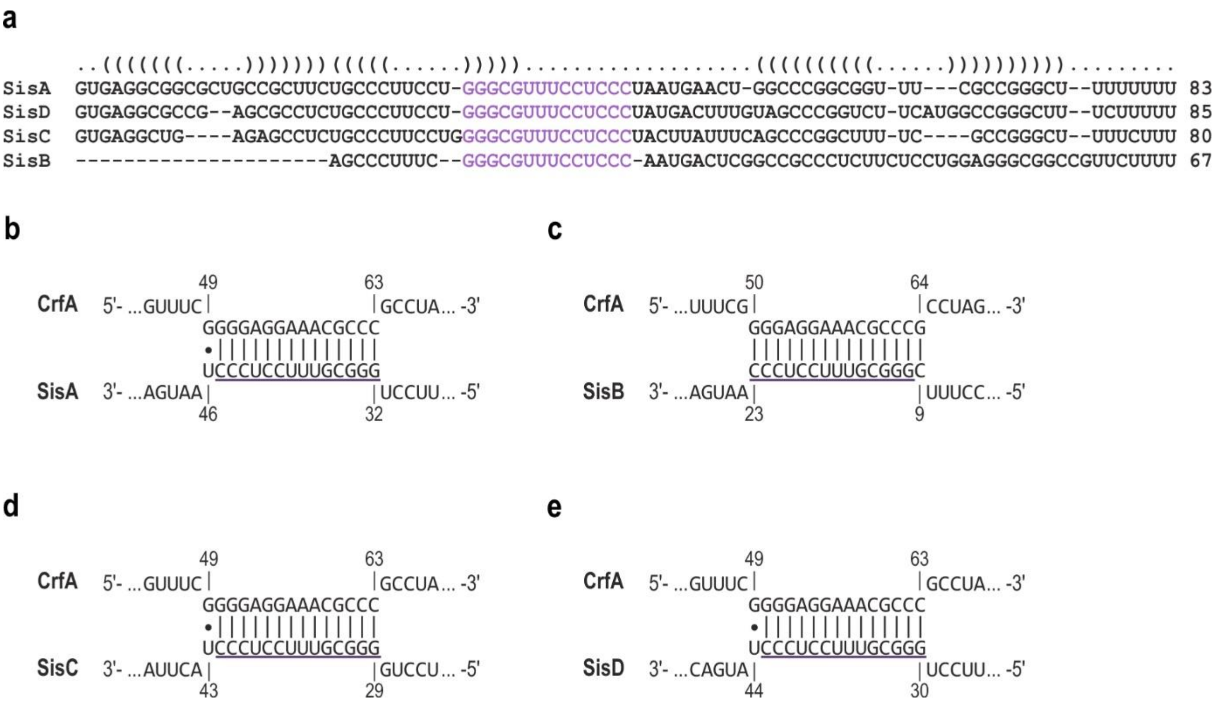

a Sequence alignment of the SisA, SisB, SisC and SisD sRNAs of *C. crescentus*. The conserved sequence stretch shared by all four sRNAs is highlighted in purple. The length of each sRNA is indicated at the end of each sequence. The predicted secondary structure of SisA is indicated above the alignment. **b-e** Base-pairing interaction between CrfA and SisA, SisB, SisC and SisD, respectively, as predicted based on RIL-seq analysis and the IntaRNA algorithm [1]. Positions are numbered relative to the TSS and the shared sequence stretch of the SisA-D family is underlined purple.

Supplementary Figure S6 - Mapping of CrfA footprint on SisA.

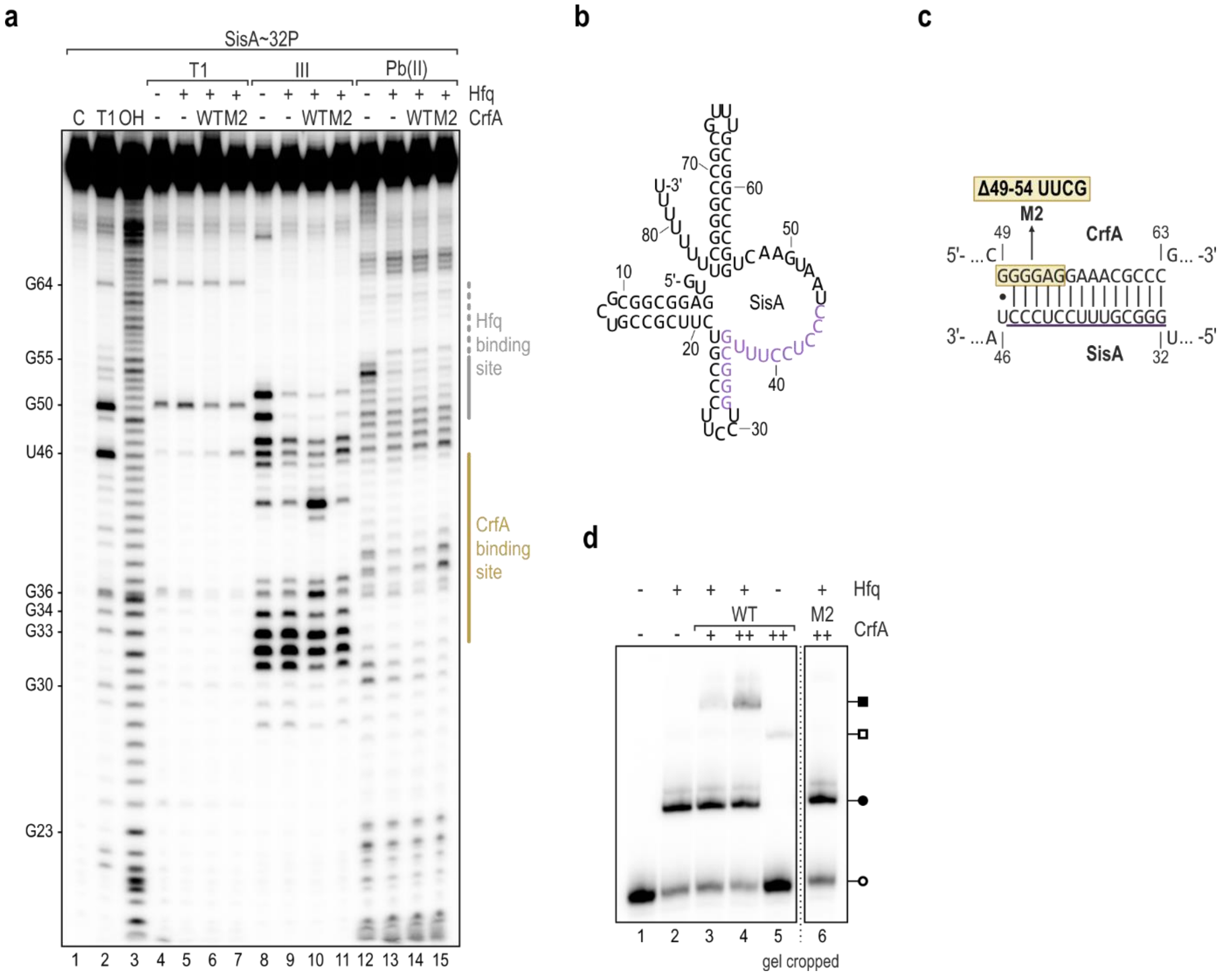

a *In vitro* structure probing of 5’-end labelled SisA sRNA (0.4 pmol) with RNase T1 (lanes 4-7), RNase III (lanes 8-11) and lead(II) (lanes 12-15) in the absence or presence (2 pmol) of purified Hfq protein and CrfA or CrfA-M2 sRNA (4 pmol), respectively. RNase T1 and alkaline (OH) ladders of SisA (lanes 2 and 3) were used to map cleavage fragments, and positions of mapped G-residues are marked relative to the TSS. The CrfA binding site indicated by CrfA footprints is marked with a yellow bar, the putative Hfq binding site is indicated with a grey bar. **b** Secondary structure of SisA as predicted using the RNAfold WebServer. The conserved sequence stretch is highlighted in purple. Positions are numbered relative to the transcriptional start site (TSS). **c** Predicted interaction between CrfA and SisA. Positions relative to the TSS are indicated and the nucleotide substitution CrfA-M2 is marked. The conserved sequence core of the SisA-D family is underlined in purple. **d** Native gel electrophoretic mobility shift assay (EMSA) using in vitro synthesized, 5’-end labelled SisA sRNA (4 nM) in the absence or presence (20 nM) of purified Hfq protein and CrfA or CrfA-M2 sRNA (+ = 4 nM, ++ = 40 nM), respectively. Open circle indicates free SisA sRNA, black circle indicates SisA-Hfq complexes, open square indicates SisA-CrfA complexes, black square indicates tripartite SisA-CrfA-Hfq complexes. Source data for this figure are provided as a Source Data file.

Supplementary Figure S7 – Modulation of CrfA levels by SisA *in vivo*.

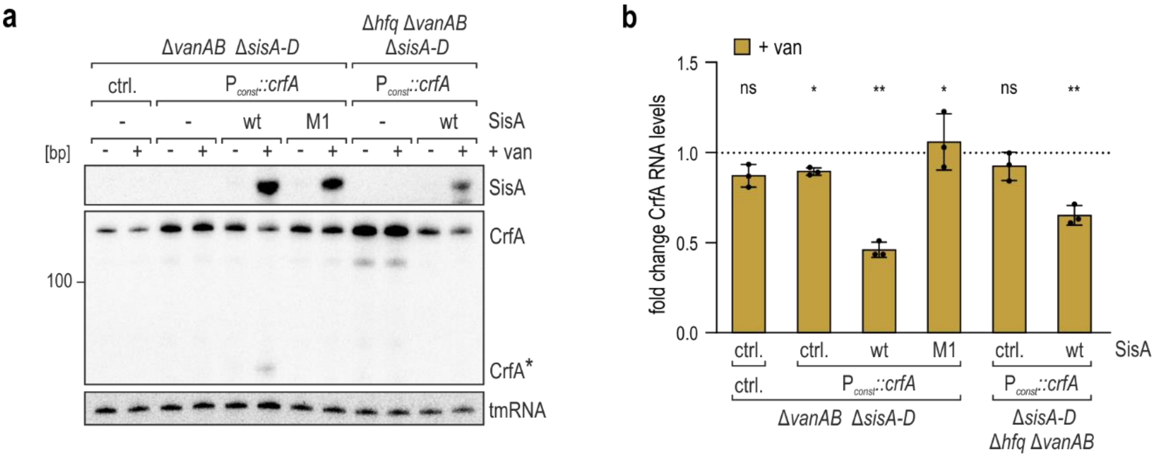

a Northern blot analysis of CrfA levels in response to pulse over-expression of SisA variants. *C. crescentus* Δ*sisA-D* Δ*vanAB* or Δ*hfq* Δ*sisA-D* Δ*vanAB* with a chromosomal construct to constitutively express CrfA (P_{const}::*crfA*) or empty vector construct (ctrl.) and carrying either an empty control vector (pBVMCS-6; ctrl.) or plasmid pP_{van}-SisA expressing SisA wild-type (wt) or SisA-M1 (M1) were grown to OD_{660} of 0.5 in PYE. Total RNA collected before (-van) and 10 min after (+van) addition of vanillate was analysed by Northern blot. tmRNA served as loading control. **b** CrfA levels after induction of SisA variants were quantified relative to the time point before induction of the respective construct. Levels are presented as mean values ± SD for three biological replicates (*n* = 3). Statistical significance was assessed using unpaired two-sided Welch's t-tests (ns for $p > 0.05$, * for $p \leq 0.05$, ** for $p \leq 0.01$). Source data for this figure are provided as a Source Data file.

Supplementary Figure S8 – Overexpression of CrfA, SisA and SisA-M2.

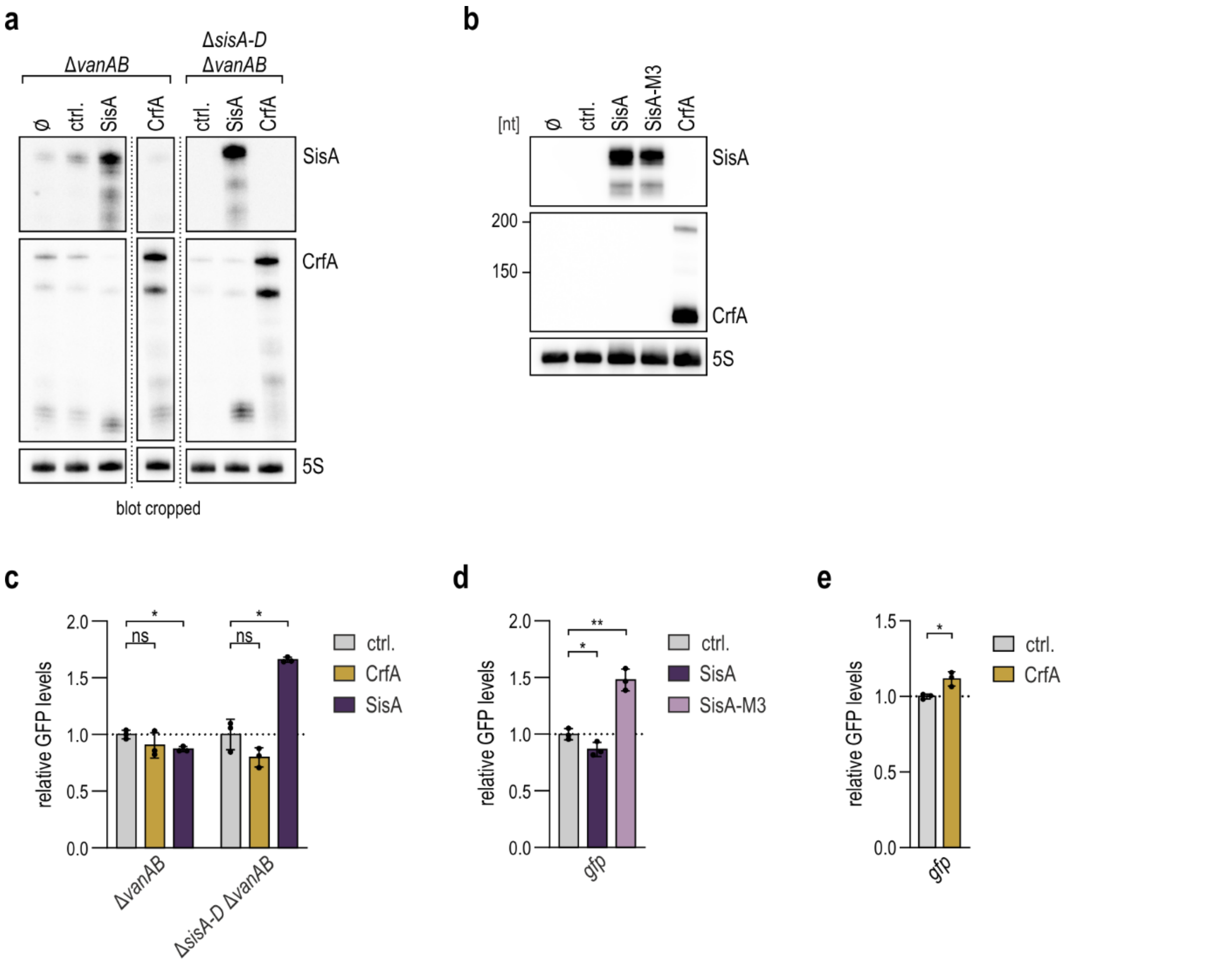

a Expression of SisA and CrfA in *C. crescentus* Δ*vanAB* and ΔΔΔ*sisA-D* Δ*vanAB* cells carrying either no plasmid (∅), empty control plasmid pBVMCS-6 (ctrl.), pP$_{van}$-SisA or pP$_{van}$-CrfA (see also Fig. S2a). RNA was extracted from cells grown in PYE in the presence of vanillate to OD$_{660}$ of 1.0 and analysed by Northern blot. 5S rRNA served as loading control. **b** Expression of SisA in *E. coli* MC4100 cells with *C. crescentus* Hfq carrying either no plasmid (∅), empty control plasmid pKP8-35 (ctrl.), pP$_{BAD}$-SisA, pP$_{BAD}$-SisA-M3, or pP$_{BAD}$-CrfA-ribozyme. RNA was extracted from cells grown in LB in the presence of arabinose for 6 h and analysed by Northern blot. 5S rRNA served as loading control. **c** Regulation of a *gfp* control construct by CrfA or SisA in *C. crescentus* Δ*vanAB* and ΔΔΔ*sisA-D* Δ*vanAB*. GFP expression was quantified by fluorescence intensity measurements. **d** Regulation of a *gfp* control construct by SisA or SisA-M3 in *E. coli* MC4100 expressing *C. crescentus* Hfq. GFP expression was quantified by fluorescence intensity measurements. **e** Regulation of a *gfp* control construct by CrfA in *E. coli* MC4100 expressing *C. crescentus* Hfq. GFP expression was quantified by fluorescence intensity measurements. **c, d, e** GFP levels were calculated as described in Fig. 1e as mean values ± SD of three biological replicates (n = 3). Statistical significance was assessed using unpaired two-sided Welch's t-tests (ns for $p > 0.05$, * for $p \leq 0.05$, ** for $p \leq 0.01$). Source data for this figure are provided as a Source Data file.

Supplementary Figure S9 – Pulse overexpression of SisA and CrfA.

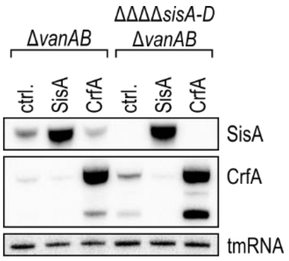

C. crescentus Δ*vanAB* or Δ*sisA-D* Δ*vanAB* carrying either an empty control vector (ctrl.; pBVMCS-6) or the expression plasmids pP_{van}-SisA or pP_{van}-CrfA were grown in biological triplicates in M2G to OD_{660} of 0.3. Total RNA was prepared from cells collected 15 min after addition of vanillate. Expression of SisA and CrfA was determined by Northern blot analysis. tmRNA served as loading control. Source data for this figure are provided as a Source Data file.

Supplementary Figure S10 - SisA target validation.

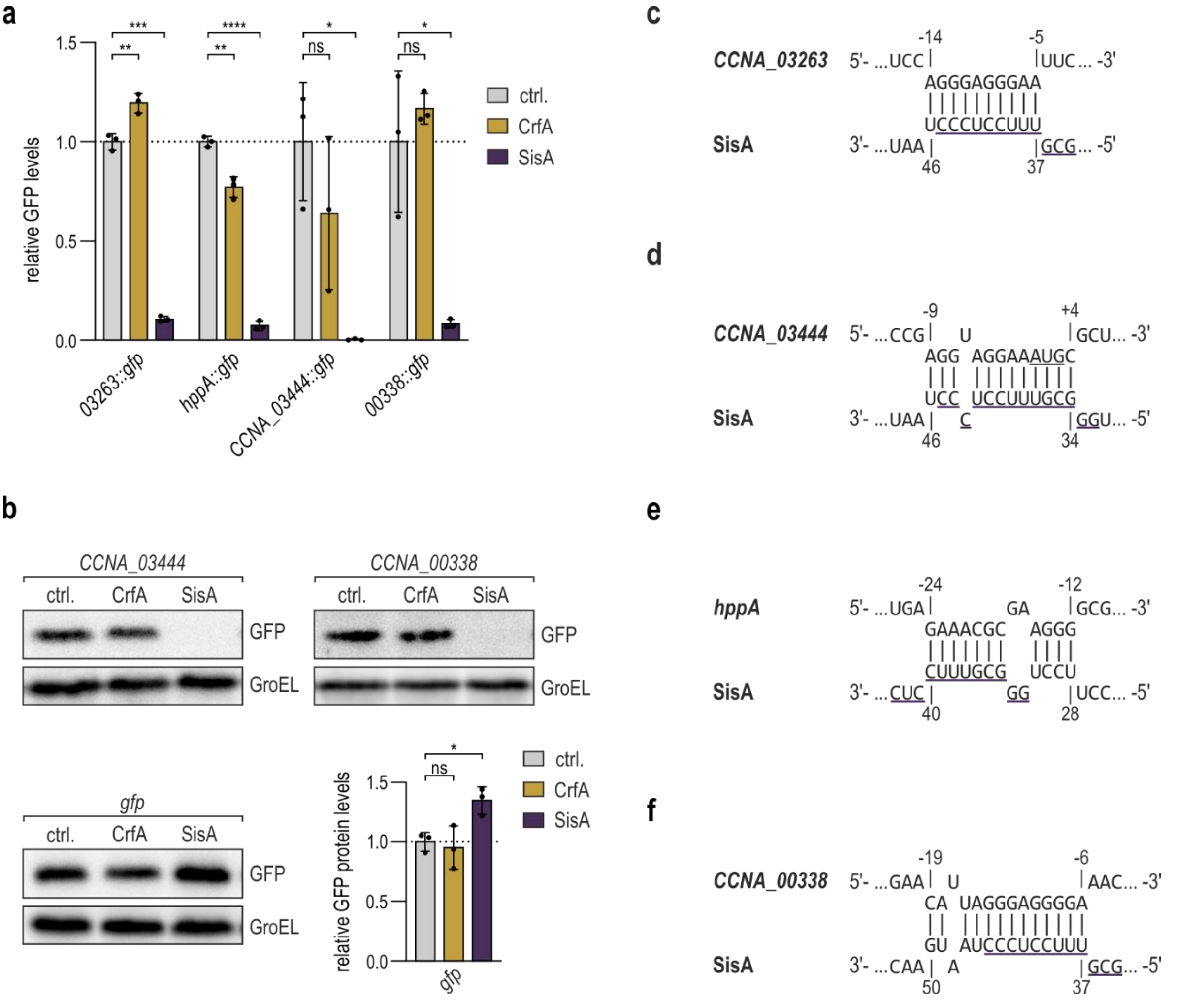

a *C. crescentus* Δ*vanAB* Δ*sisA-D* cells carrying the indicated *gfp* reporter fusion in combination with either an empty control vector (pBVMCS-6; ctrl.) or the expression plasmid pP_{van}-SisA or pP_{van}-CrfA, respectively, were grown over-night in the presence of vanillate. GFP expression was quantified either by fluorescence intensity measurements (*CCNA_03263*; *hppA*) or by Western blot analysis (*CCNA_03444*; *CCNA_00338*) of total protein samples as described in Fig. 1. **b** Western blot analysis of total protein samples collected as described in **a**. GroEL was used as loading control. GFP expression of the *gfp* control was quantified as described in Fig. 1. **a, b** GFP levels were calculated as described in Fig. 1 as mean values ± SD of three biological replicates (n = 3). Statistical significance was assessed using unpaired two-sided Welch's t-tests (ns for $p > 0.05$, * for $p \leq 0.05$, ** for $p \leq 0.01$, *** for $p \leq 0.001$, **** for $p \leq 0.0001$). **c-f** Base-pairing interaction between SisA and the indicated target mRNA as predicted based on RIL-seq analysis and the IntaRNA algorithm [1]. Nucleotide positions in the sRNA are numbered relative to the TSS, in the *CCNA_03574* mRNA relative to the start codon (underlined). The conserved sequence stretch of the SisA-D family is marked in purple. Source data for this figure are provided as a Source Data file.

Supplementary Figure S11 - CrfA-dependent modulation of SisA target genes during carbon starvation.

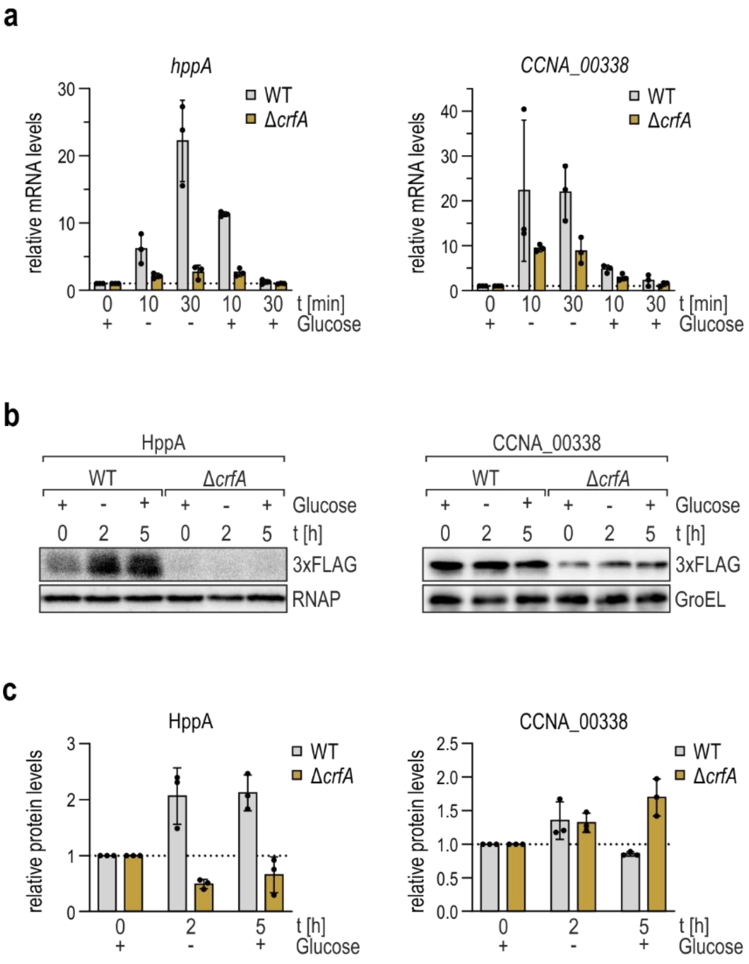

a SisA target gene mRNA levels of *hppA* and *CCNA_00338* during starvation were quantified by RT-qPCR on total RNA collected from *C. crescentus* wild-type or Δ*crfA* grown as described in Fig. 6a. Transcript levels were calculated relative to samples collected at 0 min; error bars indicate standard deviation of three biological replicates (*n* = 3). **b** Protein levels of HppA during starvation were analysed by Western blot using total protein samples collected from *C. crescentus* wild-type or Δ*crfA* grown as described in Fig. 6a. **c** Protein levels were quantified relative to the protein abundance prior to starvation in the wild-type; error bars indicate standard deviation of three biological replicates (*n* = 3). Source data underlying this figure are provided as a Source Data file.

Supplementary Table S1 – Oligonucleotides

oligo ID	sequence 5' to 3'	description
KFO-0007	GCACGGCGTCACACTTTGCT	sequencing of plasmids with backbone pBAD
KFO-0008	GACCACCGCGCTACTGCC	sequencing of plasmids with backbone pBAD
KFO-0054	CCCACATGTTAGCGCTACCAAG	sequencing of plasmids with backbone pXGFPC-4
KFO-0059	CGAATTCGTGGATCCAGATATC	amplification of pNPTs138
KFO-0060	CTTCGGCCGTGACGCGTCT	amplification of pNPTs138
KFO-0113	CAGGGGGACTTAACGACCGAGTTC	oligo probe for 5S ribosomal RNA
KFO-0141	GTTTTTCTAGATATGGGGACTGGGCCCCG	construction of plasmid pKF348-1
KFO-0144	GGATCCAATCTTGATCGTAAT	amplification of pBVMCS-6
KFO-0145	P~GTGAGGCGGCGCTGCCG	construction of plasmid pKF348-1
KFO-0169	GATATCTGGATCCACGAATTCGCCGCGCTCGCGCTCCTGGTC	construction of plasmid pKF357-5
KFO-0170	ATGGGGACTGGGCCCCGGTTAGAGTGCGGCGCAAAATTAG	construction of plasmid pKF357-5
KFO-0171	CTAATTTTGCGCCGCACTCTAACCGGGGCCCAGTCCCCAT	construction of plasmid pKF357-5
KFO-0172	AGACGCGTCACGGCCGAAGCGACGGGCGGCAGGGCGA	construction of plasmid pKF357-5
KFO-0177	GATATCTGGATCCACGAATTCGGCGAAGGTCAGCGCCCGGTC	construction of plasmid pKF359-3
KFO-0178	GCAGTCTAAGCCCCAGATCGTAGCGCTAACACAGGTCCAG	construction of plasmid pKF359-3
KFO-0179	CTGGACCTGTGTTAGCGCTACGATCTGGGGCTTAGACTGC	construction of plasmid pKF359-3
KFO-0180	AGACGCGTCACGGCCGAAGACATCATCGGCGAGGCCAGC	construction of plasmid pKF359-3
KFO-0198	TCCACTAGTTCTAGAGCGGC	amplification of pBVMCS-6
KFO-0315	TCCAGACCTACCAGTTCTTCAC	RT-qPCR oligo for *rsaA* (control)
KFO-0316	CCTGAGCGAACTTCGAGTAGTA	RT-qPCR oligo for *rsaA* (control)
KFO-0359	ACCCGCCAGGTGAACAGTC	sequencing of plasmids with backbone pGFPC-2
KFO-0513	GTTTTTTTTTTAATACGACTCACTATAGGTGAGGCGGCGCTGCC	amplification of SisA DNA template for T7 *in vitro* transcription
KFO-0514	AAAAAAAAGCCCGGCGAAACCG	amplification of SisA DNA template for T7 *in vitro* transcription
KFO-0554	GTCTAGTCTCTCATGCCGC	oligo probe for RusT RNA
KFO-0563	GTTTTTTTTTTAATACGACTCACTATAGGTGAGGCGCCGAGCGC	amplification of SisD DNA template for T7 *in vitro* transcription
KFO-0564	AAAAAGAAAGCCCGGCCATG	amplification of SisD DNA template for T7 *in vitro* transcription
KFO-0565	GTTTTTTTTTTAATACGACTCACTATAGGTGAGGCTGAGAGCCTCT	amplification of SisC DNA template for T7 *in vitro* transcription
KFO-0566	AAAGAAAAGCCCGGCGAAAAG	amplification of SisC DNA template for T7 *in vitro* transcription
KFO-0567	GTTTTTTTTTTAATACGACTCACTATAGGAGCCCTTTCGGGCGTTTC	amplification of SisB DNA template for T7 *in vitro* transcription
KFO-0568	AAAAGAACGGCCGCCCTC	amplification of SisB DNA template for T7 *in vitro* transcription
KFO-0572	ACCGTCTCCCGATCTACTTGACCTCGCCTTGAC	construction of plasmid pKF655-5
KFO-0573	GCGAGGTCAAGTAGATCGGGAGACGGTTCGAC	construction of plasmid pKF655-5
KFO-0579	GGGCCAGTTCATTAGGGAGG	oligo probe for SisA RNA
KFO-0580	GACCATGATTAGGCGAAGCTACGT	sequencing of plasmids with backbone pNTPS138
KFO-0581	TGTGCTGCAAGGCGATTAAGTTGG	sequencing of plasmids with backbone pNTPS138

KFO-0635	CCTAGGACTGAGCTAGCTGTCAAAAGCTTATATAAAAACTGTTG	construction of plasmid pKF774-1
KFO-0636	CAATTCCCCTGCTCGCGCAGGCTGG	construction of plasmid pKF649-2
KFO-0637	ATCAGCTTAGTAAAGCCCTCGCTAG	construction of plasmid pKF649-2
KFO-0668	GTTTTTCTAGAACACCAAACCCGCCGCGG	construction of plasmids pKF480-7 and pKF668-4
KFO-0684	CTTAGTCTAGATTGACAGCTAGCTCAGTC	construction of plasmid pKF777-2
KFO-0712	P~GCAAGGACGAAACGAGCC	construction of plasmids pKF480-7 and pKF668-4
KFO-0937	GTTTTTTTTAATACGACTCACTATAGGGAGGCAAGGACGAAACGAGCC	amplification of CrfA DNA template for T7 *in vitro* transcription
KFO-0938	ACACCAAACCCGCCGCGG	amplification of CrfA DNA template for T7 *in vitro* transcription
KFO-1019	GGGAATTCAATGTCGCAAACTC	RT-qPCR oligo for *CCNA_03263*
KFO-1020	GATGACGGTCGTGTTCTGTT	RT-qPCR oligo for *CCNA_03263*
KFO-1196	CTAGCGAGGGCTTTACTAAGCTGATTTAGAGTGCGGCGCAAAATTAG	construction of plasmid pKF649-2
KFO-1197	CCAGCCTGCGCGAGCAGGGGAATTGCCGGGGCCCAGTCCCCAT	construction of plasmid pKF649-2
KFO-1202	GATATCTGGATCCACGAATTCGAAGTCGCCCATCGCCAGC	construction of plasmid pKF655-5
KFO-1203	AGACGCGTCACGGCCGAAGTCGCGATGCGTCTCTGATC	construction of plasmid pKF655-5
KFO-1236	GATATCTGGATCCACGAAGCCGTGGTCGGAGGCTCTGC	construction of plasmid pKF654-5
KFO-1237	TCGAGCGCGAACGCCAAGAGCCGCCTTGAA	construction of plasmid pKF654-5
KFO-1238	TTCAAGGCGGCTCTTGGCGTTCGCGCTCGA	construction of plasmid pKF654-5
KFO-1239	AGACGCGTCACGGCCGAACAAGGAGAACATCCTCGTCAACG	construction of plasmid pKF654-5
KFO-1280	CTTTTGAATTCATGACACTGTTCCGGGTGTCATTT	construction of plasmid pKF656-1
KFO-1281	CTTTTGGTACCGGCGCCGTACAGCACCGC	construction of plasmid pKF656-1
KFO-1282	CTTTTGAATTCGCGTCGGGAGTGGCAACT	construction of plasmid pKF657-2
KFO-1283	CTTTTGGTACCAGAGAGCGCGCTGGCGCC	construction of plasmid pKF657-2
KFO-1284	CTTTTGAATTCATCCATTCGAACACTCGTTCGGCG	construction of plasmid pKF658-2
KFO-1285	CTTTTGGTACCGCCAAGACCGGCGAGGAC	construction of plasmid pKF658-2
KFO-1298	TCTCTGGTGGGCTCGTTTCGTCCTTGCAGT	oligo probe for CrfA RNA
KFO-1306	TTTCTTCGGAAACGCCCGCCTAGATCG	construction of plasmid pKF666-1 and pKF769-1
KFO-1307	TTTCCGAAGAAACGCCGAAGAACGGGAG	construction of plasmid pKF666-1 and pKF769-1
KFO-1308	GATATCTGGATCCACGAATTCGTGATCCTGAAGAGCCAGGC	construction of plasmid pKF667-1
KFO-1309	CTAGCGAGGGCTTTACTAAGCTGATTGACGGTGTCAACGCAGCC	construction of plasmid pKF667-1
KFO-1310	CCAGCCTGCGCGAGCAGGGGAATTGCACAAATTTGACGGAAGAGTC	construction of plasmid pKF667-1
KFO-1311	AGACGCGTCACGGCCGAAGTGGTCTGGGTGGCGCTG	construction of plasmid pKF667-1
KFO-1371	GAGATACTGAGCACATGCATGCGTCGGGAGTGGCAACT	construction of plasmid pKF682-1
KFO-1372	CCAGCGGATCCGCTAGCAGAGAGCGCGCTGGCGCC	construction of plasmid pKF682-1
KFO-1399	CTTTTGAATTCAACAGCGTTAGGGAGCGCAT	construction of plasmid pKF691-1
KFO-1400	CTTTTGGTACCGAAGACGTCGCCGAAGCG	construction of plasmid pKF691-1
KFO-1418	GAGAAGAGGGCGGCCGAGTCATTGGGAGGA	oligo probe for SisB RNA
KFO-1419	CGAAAAGCCGGGCTGAAATAAGTAGGGAGGA	oligo probe for SisC RNA
KFO-1420	ATGAAGACCGGGCTACAAAGTCATAGGGAG	oligo probe for SisD RNA

KFO-1453	CGGCGAACTCTTCAGCGAAGTTATCGTTCGC	oligo probe for tmRNA
KFO-1544	TCTAGAGGCATCAAATAAAAC	amplification of pZE12-luc
KFO-1694	CTGGGCCCTGTTTCACAAATTTGACGGAAGAGTC	construction of plasmid pKF767-2
KFO-1695	CGTTGACACCGTCACGAATTTGACGTTTCTACACA	construction of plasmid pKF767-2
KFO-1696	CACAAATTTGACGGAAACAGGGCCCAGATACA	construction of plasmid pKF767-2
KFO-1721	AACGTCAAATTCGTGACGGTGTCAACGCAGCC	construction of plasmid pKF767-2
KFO-1780	CAGTCCTAGGTATAATGCTAGCGCAAGGACGAAACGAGC	construction of plasmid pKF774-1
KFO-1781	GTTTTCATATGACACCAAACCCGCCGC	construction of plasmid pKF777-2
KFO-1841	GTTTTTGCTAGCTTTAGGTAAAGGGTTTTTGCGAG	construction of plasmid pKF778-3
KFO-1842	GTTTTTCATATGAAAAAAAAGCCCGGCGAAACC	construction of plasmid pKF778-3
KFO-2133	CTTTTGAATTCACTGAGGGGAACCAGTGATG	construction of plasmid pKF829-1
KFO-2135	CTTTTGGTACCGGTGGCCGTGAACTGGTAG	construction of plasmid pKF829-1
KFO-2495	TTTCCTGGGTAATGAACTGGCCCGGCG	construction of plasmids pKF881-1 and pKF897-1
KFO-2496	TCATTACCCAGGAAACGCCCAGGAAGG	construction of plasmids pKF881-1 and pKF897-1
KFO-2497	GTTTCGCCCAGGAAACGCCCGCCTAGA	construction of plasmids pKF882-1
KFO-2498	GTTTCCTGGGCGAAACGCCGAAGAACGG	construction of plasmids pKF882-1
KFO-2516	GTGATCGGCGGCTTCTATT	RT-qPCR oligo for *CCNA_03574*
KFO-2517	GCTTGCCGACCGTATAGTT	RT-qPCR oligo for *CCNA_03574*
KFO-2518	GCACGTACGGCAGCTATAAT	RT-qPCR oligo for *CCNA_00338*
KFO-2519	CATAGGCCAGACGGAAGAAC	RT-qPCR oligo for *CCNA_00338*
KFO-2522	GTGCAGACAGCGAGTCTAATG	RT-qPCR oligo for *hppA*
KFO-2523	CCGGTTGCTCCCAAGATTT	RT-qPCR oligo for *hppA*
KFO-2526	CACCGCCAATCCATACGAT	RT-qPCR oligo for *CCNA_03444*
KFO-2527	GAATAGTTCGTGCCGTCCTT	RT-qPCR oligo for *CCNA_03444*
KFO-2530	GATATCTGGATCCACGAATTCGCGTCGAGGCCGAACTGACCG	construction of plasmid pKF891-1
KFO-2531	TTGTAGTCGATGTCGTGGTCCTTGTAGTCGCCGTCGTGGTCCTTGTAGTCGAAC GACTTCCTGACCGTCA	construction of plasmid pKF891-1
KFO-2532	ACTACAAGGACCACGACATCGACTACAAGGACGACGACGACAAGTAGTAAGGGT GGACAGCCTGGAA	construction of plasmid pKF891-1
KFO-2533	AGACGCGTCACGGCCGAAGGTCAGCACGGGAAAGGGGA	construction of plasmid pKF891-1
KFO-2534	GATATCTGGATCCACGAATTCGCCAAGCTGAGCGCGAACCTC	construction of plasmid pKF890-1
KFO-2535	TTGTAGTCGATGTCGTGGTCCTTGTAGTCGCCGTCGTGGTCCTTGTAGTCGAAG GCCACCTCGATCGAAG	construction of plasmid pKF890-1
KFO-2536	ACTACAAGGACCACGACATCGACTACAAGGACGACGACGACAAGTAGTAATCTA GGCGATAGCTGCTCGG	construction of plasmid pKF890-1
KFO-2537	AGACGCGTCACGGCCGAAGCGAAGGAGTAAACCGCCAGC	construction of plasmid pKF890-1
KFO-2542	GATATCTGGATCCACGAATTCGCCTATCAAGGCTTCCTGGCG	construction of plasmid pKF892-1
KFO-2543	TTGTAGTCGATGTCGTGGTCCTTGTAGTCGCCGTCGTGGTCCTTGTAGTCCCAG GACTTGGTGATCGCGA	construction of plasmid pKF892-1
KFO-2544	ACTACAAGGACCACGACATCGACTACAAGGACGACGACGACAAGTAGTAAACCC CAGGACGAGCGCCTCG	construction of plasmid pKF892-1
KFO-2545	AGACGCGTCACGGCCGAAGCGACATAGGCGGTGCGGAAG	construction of plasmid pKF892-1
KFO-2559	GAAACCGCCGGGCCAGTTCATT	oligo probe for SisA-M1 and SisA-M3 RNA variants
KFO-2562	GATATCTGGATCCACGAATTGGTCATCCAGAGCGGCTTCA	construction of plasmid pKF923-1

KFO-2563	TTGTAGTCGATGTCGTGGTCCTTGTAGTCGCCGTCGTGGTCCTTGTAGTCGAAC TTCGAGCGCAGGGTCA	construction of plasmid pKF923-1
KFO-2564	ACTACAAGGACCACGACATCGACTACAAGGACGACGACGACAAGTAGTTCTGCC GGGAGGGGGACCG	construction of plasmid pKF923-1
KFO-2565	AGACGCGTCACGGCCGAAGGGCAAGCTGCGGTCACGGCC	construction of plasmid pKF923-1
KFO-2566	GATATCTGGATCCACGAATCGAGTCGGTCGTCGCCGAG	construction of plasmid pKF918-1
KFO-2567	TTGTAGTCGATGTCGTGGTCCTTGTAGTCGCCGTCGTGGTCCTTGTAGTCGACG CCGTGGGCCAGGACCG	construction of plasmid pKF918-1
KFO-2568	ACTACAAGGACCACGACATCGACTACAAGGACGACGACGACAAGTAGTCTTTCA GAGGGCCAGTCAT	construction of plasmid pKF918-1
KFO-2569	AGACGCGTCACGGCCGAAGCGAAGGGCTGAAGATCTTTC	construction of plasmid pKF918-1
KFO-2643	CGCAACTCTCTACTGTTTCTCCGTGAGGCGGCGCTGCC	construction of plasmid pKF1014-1
KFO-2644	GTTCTGATTTAATCTAGAAAAAAAAGCCCGGCGAAACC	construction of plasmid pKF1014-1
KFO-2645	CGCAACTCTCTACTGTTTCTCCGCAAGGACGAAACGAGCCC	construction of plasmid pKF1064-1
KFO-2814	CTTTTGAATTCAACCAAAGGTCCGACAAGGA	construction of plasmid pKF971-1
KFO-2816	CTTTTGGTACCGACCTTCCCGCCGTCC	construction of plasmid pKF971-1
KFO-2951	AGGGAGCAAATCATGACCATCGTGTCGTC	construction of plasmid pKF999-1
KFO-2952	TGATTTGCTCCCTGGTGTTTTTGGCG	construction of plasmid pKF999-1
KFO-2953	CGAGGAGGCTGGGACCATGCCGGCCACACCAAACCCGCCGCG	construction of plasmid pKF996-1
KFO-2954	TCCCCTCGGTAATGGCGAATGGGACTCTAGAGGCATCAAATAAAACGAAA	construction of plasmid pKF996-1
KFO-2955	GGCCGGCATGGTCCCAGCCTCCTCGCTGGCGCCGGCTGGGCAACATTCCGAG GGGACCGTCCCCTCGGTAATGGCGAATGGGAC	construction of plasmid pKF996-1
KFO-2956	GTCCCATTCGCCATTACCGAGGGGACGGTCCCCTCGGAATGTTGCCCAGCCGG CGCCAGCGAGGAGGCTGGGACCATGCCGGCC	construction of plasmid pKF996-1
KFO-2957	CTTTTGAATTCGCGTGAGATTCCGGCCG	construction of plasmid pKF1000-1
KFO-2958	CTTTTGGTACCCGGCAGCAGCACGCCC	construction of plasmid pKF1000-1
KFO-2959	CTTTTGAATTCGTTTAACGAACGAGAGCGCC	construction of plasmid pKF1001-1
KFO-2960	CTTTTGGTACCGTCGATGAGGGCGTCGAA	construction of plasmid pKF1001-1
KFO-2983	GCGTTTGCTCCCTAATGAACTGGCCC	construction of plasmid pKF1022-1
KFO-2984	AGGGAGCAAACGCCCAGGAAGGGCA	construction of plasmid pKF1022-1
KFO-2985	CTTTTGAATTCGCGAAAAGCGGACGTAACCG	construction of plasmid pKF989-1
KFO-2986	CTTTTGGTACCCTGACCGGACGATTTTTCGCC	construction of plasmid pKF989-1
KFO-3328	GTTCTGATTTAATCTAGGTCCCATTCGCCATTACCGA	construction of plasmid pKF1064-1
KFO-3329	TCCCCTCGGTAATGGCGAATGGGACCTAGATTAAATCAGAACGCAGAAG	construction of plasmid pKF1064-1
KPO-0196	GGAGAAACAGTAGAGAGTTGCG	amplification of pKP8-35
KPO-0411	CTAGATTAAATCAGAAC	amplification of pKP8-35
KPO-1702	ATGCATGTGCTCAGTATCTCTATC	amplification of pXG10sf
KPO-2372	CAGGTAGTTTTCCAGTAGTGC	sequencing of plasmids with backbone pXG10sf
KPO-3071	TTCCGCTTCCTCGCTCAC	sequencing of plasmids with backbone pXG10sf
KPO-7614	CCTAGCGGATCCGCTGGC	amplification of pXG10sf
M13fwd	GTAAAACGACGGCCAGT	sequencing of plasmids with backbone pBVMCS-6 and pXGFPC-4
pPLlacOD	GTGCTCAGTATCTTGTTATCCG	amplification of pZE12-luc
23S-1	[Btn]ACCTTTCCCTCACGGTACTGGTTCGCTATCGGTCA	oligo for 23S rRNA depletion #1
23S-2	[Btn]AGTCGCTGGCTCATTATACAAAAGGTACGCCGTCACC	oligo for 23S rRNA depletion #2

23S-3	[Btn]TCGGGGAGAACCAGCTATCTCCGGGTTTGATTGGC	oligo for 23S rRNA depletion #3
23S-4	[Btn]GTGGCTGCTTCTAAGCCAACATCCTG	oligo for 23S rRNA depletion #4
23S-5	[Btn]GGGTACAGGAATATTAACCTGATTTCCATCGACTACGCC	oligo for 23S rRNA depletion #5
23S-6	[Btn]CACCTGTGTCGGTTTGGGGTACGGT	oligo for 23S rRNA depletion #6
23S-7	[Btn]TCGTGCGGGTCGGAACTTACCCGACAAG	oligo for 23S rRNA depletion #7
23S-8	[Btn]GAGCCGACATCGAGGTGCCAAACA	oligo for 23S rRNA depletion #8
23S-9	[Btn]CGGCGGATAGGGACCGAACTGTCTCACGAC	oligo for 23S rRNA depletion #9
16S-1	[Btn]CCGCTCGACTTGCATGTGTTAAGCATGCCGACAGCGTTCG	oligo for 16S rRNA depletion #1
16S-2	[Btn]CCCATTGTGCAAGATTCCCTACTGCTGCCTCCCGT	oligo for 16S rRNA depletion #2
16S-3	[Btn]ACCGCGGCTGCTGGCACGGAGT	oligo for 16S rRNA depletion #3
16S-4	[Btn]ACGGCGTGGACTACCAGGGTAT	oligo for 16S rRNA depletion #4
16S-5	[Btn]TCCACATGCTCCACCGCTTGTGCGGGCCCCCG	oligo for 16S rRNA depletion #5
16S-6	[Btn]ACCCAACATCTCACAACACGAGCTGACGACA	oligo for 16S rRNA depletion #6
16S-7	[Btn]GGGCAGTGTGTACAAGGCCCGGGA	oligo for 16S rRNA depletion #7
16S-8	[Btn]AAGGAGGTGATCCAGCCGCAG	oligo for 16S rRNA depletion #8
23S-GN1	[Btn]CACGTCCTTCATCGCCTTTTACTGCCAAGGCATCC	oligo for 23S rRNA depletion Gram Neg #1
23S-GN2	[Btn]CCACACCCGGCCTATCAACGTGGTGGTCTTCGACG	oligo for 23S rRNA depletion Gram Neg #2
Cc5S-1	[Btn]CCGAGTTCGGAATGGGATCGGGTGGG	oligo for *C. crescentus* 5S rRNA depletion #1
Cc5S-2	[Btn]CTTGAGACGAAGTACCATTGGCCCAGGG	oligo for *C. crescentus* 5S rRNA depletion #2

Supplementary Table S2 – Plasmids

plasmid ID	description	backbone/marker	reference
pBVMCS-6	empty vector	pBVMCS-6/CmR	[2]
pXGFPC-4	empty vector	pXGFPC-4/GentR	[2]
pNPTS138	empty vector	pNPTS138/KanR	M. R. Alley, unpublished
pXG10sf	empty vector	pXG10sf/CmR	[3]
pZE12-luc	empty vector	pZE12-luc/AmpR	[4]
pKF348-1	expression of *sisA* under control of the *van* promotor (P_{van})	pBVMCS-6/CmR	[5]
pKF357-5	allelic replacement of *sisA*	pNPTS138/KanR	this study
pKF359-3	allelic replacement of *sisD*	pNPTS138/KanR	this study
pKF384-1	expression of *gfp* under control of the *rsaA* promoter; integration into *rsaA* locus	pGFPC-2/KanR	[6]
pKF385-2	expression of *rsaA::gfp* translational fusion (up to +45 of *rsaA* relative to the translational start site) under control of the *rsaA* promoter; integration into *rsaA* locus	pGFPC-2/KanR	[6]
pKF480-7	expression of *crfA* under control of the *van* promotor (P_{van})	pBVMCS-6/CmR	[5]
pKF485-1	empty vector, integration upstream of the *xylX* locus	pXGFPC-4/GentR	this study
pKF494-2	expression of *CCNA_03263::gfp* translational fusion (-116 to +60 of *CCNA_03263* relative to the translational start site) under control of the *rsaA* promotor, integration into *rsaA* locus; insert amplified with KFO-0715/KFO-0716	pGFPC-2/KanR	[7]
pKF546-1	expression of *CCNA_00338::gfp* translational fusion (-94 to +60 of *CCNA_00338* relative to the translational start site) under control of the *rsaA* promotor, integration into *rsaA* locus; insert amplified with KFO-0926/KFO-0927	pGFPC-2/KanR	[7]
pKF649-2	allelic replacement of *sisA* with *omega* cassette (StrepR/SpecR)	pNPTS138/KanR	this study
pKF654-5	allelic replacement of *sisC*	pNPTS138/KanR	this study
pKF655-5	allelic replacement of *sisB*	pNPTS138/KanR	this study
pKF656-1	expression of *hppA::gfp* translational fusion (-155 to +60 of *hppA* relative to the translational start site) under control of the *rsaA* promotor, integration into *rsaA* locus; insert amplified with KFO-1280/KFO-1281	pGFPC-2/KanR	this study
pKF657-2	expression of CCNA_*03574::gfp* translational fusion (-50 to +60 of *CCNA_03574* relative to the translational start site) under control of the *rsaA* promotor, integration into *rsaA* locus; insert amplified with KFO-1282/KFO-1283	pGFPC-2/KanR	this study
pKF658-2	expression of CCNA_*03444::gfp* translational fusion (-85 to +60 of *CCNA_03444* relative to the translational start site) under control of the *rsaA* promotor, integration into *rsaA* locus; insert amplified with KFO-1284/KFO-1285	pGFPC-2/KanR	this study
pKF666-1	expression of *crfA-M2* under control of the *van* promotor (P_{van})	pBVMCS-6/CmR	this study
pKF667-1	allelic replacement of *crfA* with *omega* cassette (StrepR/SpecR)	pNPTS138/KanR	this study
pKF668-4	expression of *crfA* under control of the constitutive promotor P_{LlacO} in *E. coli*	pZE12-luc/AmpR	this study
pKF682-1	expression of *CCNA_03574::gfp* translational fusion (-52 to +60 of *CCNA_03574* relative to the translational start site) under control of the constitutive PLtetO-1 promotor in *E. coli*; insert amplified with KFO-1282/KFO-1283	pXG10sf/CmR	this study
pKF691-1	expression of CCNA_*02357::gfp* translational fusion (-21 to +60 of *CCNA_02357* relative to the translational start site) under control of the *rsaA* promotor, integration into *rsaA* locus; insert amplified with KFO-1399/KFO-1400	pGFPC-2/KanR	this study
pKF767-2	allelic replacement of *crfA* with *crfA*, template plasmid for pKF769-1	pNPTS138/KanR	this study
pKF769-1	allelic replacement of *crfA* with *crfA-M2*	pNPTS138/KanR	this study
pKF774-1	expression of crfA under control of the constitutive promotor J23119 (P_{const})	pBVMCS-6/CmR	this study
pKF777-2	expression of *crfA* under control of the constitutive promotor J23119 (P_{const}), integration upstream of the *xylX* locus	pXGFPC-4/GentR	this study
pKF778-3	expression of *sisA* under control of its native promotor P_{sisA}, integration upstream of the *xylX* locus	pXGFPC-4/GentR	this study

pKF829-1	expression of *xylX::gfp* translational fusion (-62 to +60 of *xylX* relative to the translational start site) under control of the *rsaA* promotor, integration into *rsaA* locus; insert amplified with KFO-2133/KFO-2135	pGFPC-2/KanR	this study
pKF881-1	expression of *sisA-M1* under control of the *van* promotor (P_{van})	pBVMCS-6/CmR	this study
pKF882-1	expression of *crfA-M1* under control of the *van* promotor (P_{van})	pBVMCS-6/CmR	this study
pKF890-1	chromosomal integration of 3XFLAG at *CCNA_00338* C-terminus via allelic replacement	pNPTS138/KanR	this study
pKF891-1	chromosomal integration of 3XFLAG at *CCNA_03574* C-terminus via allelic replacement	pNPTS138/KanR	this study
pKF892-1	chromosomal integration of 3XFLAG at *CCNA_03263* C-terminus via allelic replacement	pNPTS138/KanR	this study
pKF897-1	expression of *sisA-M1* under control of its native promotor P_{sisA}, integration upstream of the *xylX* locus	pXGFPC-4/GentR	this study
pKF918-1	chromosomal integration of 3XFLAG at *CCNA_01425* C-terminus via allelic replacement	pNPTS138/KanR	this study
pKF923-1	chromosomal integration of 3XFLAG at *CCNA_03444* C-terminus via allelic replacement	pNPTS138/KanR	this study
pKF971-1	expression of CCNA_*00857::gfp* translational fusion (-67 to +60 of *CCNA_00857* relative to the translational start site) under control of the *rsaA* promotor, integration into *rsaA* locus; insert amplified with KFO-2814/KFO-2816	pGFPC-2/KanR	this study
pKF989-1	expression of CCNA_*00543::gfp* translational fusion (-35 to +60 of *CCNA_00543* relative to the translational start site) under control of the *rsaA* promotor, integration into *rsaA* locus; insert amplified with KFO-2985/KFO-2986	pGFPC-2/KanR	this study
pKF996-1	expression of *crfA*-ribozyme under control of the constitutive promotor P_{LlacO} in *E. coli*	pZE12-luc/AmpR	this study
pKF999-1	expression of *CCNA_03574-M3::gfp* translational fusion (-52 to +60 of *CCNA_03574* relative to the translational start site, SNE G-6C) under control of the constitutive PLtetO-1 promotor in *E. coli*; pKF682-1 amplified with KFO-2951/KFO-2952 to introduce SNE	pXG10sf/CmR	this study
pKF1000-1	expression of CCNA_*02914::gfp* translational fusion (-70 to +60 of *CCNA_02914* relative to the translational start site) under control of the *rsaA* promotor, integration into *rsaA* locus; insert amplified with KFO-2957/KFO-2958	pGFPC-2/KanR	this study
pKF1001-1	expression of CCNA_*01807::gfp* translational fusion (-48 to +60 of *CCNA_01807* relative to the translational start site) under control of the *rsaA* promotor, integration into *rsaA* locus; insert amplified with KFO-2959/KFO-2960	pGFPC-2/KanR	this study
pKF1014-1	expression of *sisA* under control of the inducible promotor P_{BAD} in *E. coli*	pBAD5A/AmpR	this study
pKF1022-1	expression of *sisA-M3* under control of the inducible promotor P_{BAD} in *E. coli*	pBAD5A/AmpR	this study
pKF1064-1	expression of *crfA*-ribozyme under control of the inducible promotor P_{BAD} in *E. coli*	pBAD5A/AmpR	this study
pKP8-35	pBAD control plasmid	pBAD5A/AmpR	[8]

Supplementary Table S3 – Bacterial strains

strain	stock name	bacterium	genotype/relevant markers	source/reference
wild type	KFS-0006	*C. crescentus NA1000*		laboratory stock
ΔvanAB	KFS-0058	*C. crescentus NA1000*	*ΔvanAB*	[6]
3XFLAG::hfq	KFS-0344	*C. crescentus NA1000*	*3XFLAG::hfq*	[6]
	KFS-0537	*C. crescentus NA1000*	*ΔsisD ΔvanAB*	this study
Δhfq	KFS-0570	*C. crescentus NA1000*	*Δhfq::TetR*	[9]
	KFS-606	*C. crescentus NA1000*	*ΔvanAB xylX::pKF485-1*	this study
Δhfq ΔvanAB	KFS-0916	*C. crescentus NA1000*	*Δhfq::TetR ΔvanAB*	this study
	KFS-1496	*C. crescentus NA1000*	*ΔsisB ΔsisD ΔvanAB*	this study
	KFS-1506	*C. crescentus NA1000*	*ΔsisB ΔsisC ΔsisD ΔvanAB*	this study
	KFS-1508	*C. crescentus NA1000*	*ΔcrfA::Ω ΔvanAB*	this study
ΔcrfA	KFS-1547	*C. crescentus NA1000*	*ΔcrfA::Ω*	this study
ΔsisA-D ΔvanAB	KFS-1707	*C. crescentus NA1000*	*ΔsisA::Ω ΔsisB ΔsisC ΔsisD ΔvanAB*	this study
	KFS-1737	*C. crescentus NA1000*	*crfA-M2*	this study
ΔsisA::Ω ΔvanAB	KFS-1741	*C. crescentus NA1000*	*ΔsisA::Ω ΔvanAB*	this study
	KFS-1756	*C. crescentus NA1000*	*ΔsisA::Ω ΔvanAB xylX::pKF485-1*	this study
	KFS-1758	*C. crescentus NA1000*	*ΔsisA::Ω ΔsisB ΔsisC ΔsisD ΔvanAB xylX::pKF485-1*	this study
ΔvanAB ΔsisA-D P_{const}*-crfA*	KFS-1759	*C. crescentus NA1000*	*ΔsisA::Ω ΔsisB ΔsisC ΔsisD ΔvanAB xylX::pKF777-2*	this study
	KFS-2108	*C. crescentus NA1000*	*CCNA_03574::3xFLAG*	this study
Δhfq ΔvanAB ΔsisA-D P_{const}*-crfA*	KFS-2111	*C. crescentus NA1000*	*Δhfq::TetR ΔsisA::Ω ΔsisB ΔsisC ΔsisD ΔvanAB xylX::pKF777-2*	this study
	KFS-2113	*C. crescentus NA1000*	*CCNA_00338::3xFLAG*	this study
	KFS-2114	*C. crescentus NA1000*	*CCNA_03263::3xFLAG*	this study
	KFS-2126	*C. crescentus NA1000*	*ΔcrfA::Ω CCNA_03574::3xFLAG*	this study

	KFS-2127	*C. crescentus NA1000*	Δ*crfA::Ω CCNA_00338::3xFLAG*	this study
	KFS-2128	*C. crescentus NA1000*	Δ*crfA::Ω CCNA_03263::3xFLAG*	this study
Δ*sisA::Ω* ∆*vanAB* P_{sisA}-*sisA*	KFS-2132	*C. crescentus NA1000*	Δ*sisA::Ω* ∆*vanAB xylX::pKF778-3*	this study
Δ*sisA::Ω* ∆*vanAB* P_{sisA}-*sisA-M1*	KFS-2133	*C. crescentus NA1000*	Δ*sisA::Ω* ∆*vanAB xylX::pKF897-1*	this study
	KFS-2209	*C. crescentus NA1000*	*CCNA_01425::3xFLAG*	this study
	KFS-2221	*C. crescentus NA1000*	Δ*crfA::Ω CCNA_01425::3xFLAG*	this study
	KFS-2229	*C. crescentus NA1000*	*CCNA_03444::3xFLAG*	this study
	KFS-2238	*C. crescentus NA1000*	Δ*crfA::Ω CCNA_03444::3xFLAG*	this study
E. coli TOP10	KFS-0088	*E. coli*	*F- mcrA Δ(mrr-hsdRMS-mcrBC) Φ80lacZΔM15 ΔlacX74 recA1 araD139 Δ(ara-leu)7697 galU galK rpsL endA1 nupG λ-*	Invitrogen
E. coli MC4100 *Cchfq*	KFS-0706	*E. coli MC4100*	*Phfq::CChfq*	[10]

Supplementary References

1. Wright, P. R. *et al.* Comparative genomics boosts target prediction for bacterial small RNAs. *Proc Natl Acad Sci U S A* **110**, E3487-3496, doi:10.1073/pnas.1303248110 (2013).
2. Thanbichler, M., Iniesta, A. A. & Shapiro, L. A comprehensive set of plasmids for vanillate- and xylose-inducible gene expression in Caulobacter crescentus. *Nucleic Acids Research* **35**, e137-e137, doi:10.1093/nar/gkm818 (2007).
3. Corcoran, C. P. *et al.* Superfolder GFP reporters validate diverse new mRNA targets of the classic porin regulator, MicF RNA. *Molecular Microbiology* **84**, 428-445, doi:https://doi.org/10.1111/j.1365-2958.2012.08031.x (2012).
4. Lutz, R. & Bujard, H. Independent and tight regulation of transcriptional units in *Escherichia coli* via the LacR/O, the TetR/O and AraC/I1-I2 regulatory elements. *Nucleic Acids Res* **25**, 1203-1210 (1997).
5. Velasco-Gomariz, M., Sulzer, J., Faber, F. & Fröhlich, K. S. An sRNA overexpression library reveals AbnZ as a negative regulator of an essential translocation module in Caulobacter crescentus. *Nucleic Acids Res* **53**, doi:10.1093/nar/gkae1139 (2025).
6. Fröhlich, K. S., Förstner, K. U. & Gitai, Z. Post-transcriptional gene regulation by an Hfq-independent small RNA in Caulobacter crescentus. *Nucleic Acids Research* **46**, 10969-10982, doi:10.1093/nar/gky765 (2018).
7. Vogt, L. N. *et al.* Genome-wide profiling of Hfq-bound RNAs reveals the iron-responsive small RNA RusT in *Caulobacter crescentus*. *mBio* **15**, e03153-03123, doi:doi:10.1128/mbio.03153-23 (2024).
8. Papenfort, K. *et al.* σE-dependent small RNAs of Salmonella respond to membrane stress by accelerating global omp mRNA decay. *Molecular Microbiology* **62**, 1674-1688, doi:https://doi.org/10.1111/j.1365-2958.2006.05524.x (2006).
9. Irnov, I. *et al.* Crosstalk between the tricarboxylic acid cycle and peptidoglycan synthesis in Caulobacter crescentus through the homeostatic control of α-ketoglutarate. *PLOS Genetics* **13**, e1006978, doi:10.1371/journal.pgen.1006978 (2017).
10. Santiago-Frangos, A. *et al.* Caulobacter crescentus Hfq structure reveals a conserved mechanism of RNA annealing regulation. *Proceedings of the National Academy of Sciences* **116**, 10978-10987, doi:10.1073/pnas.1814428116 (2019).
