# Supplementary material for: An RNA sponge directs the transition from feast to famine in Caulobacter crescentus
Source: Nat Commun. 2025 Oct 27;16:9478. doi: 10.1038/s41467-025-65274-1 (PMC12559287; doi:10.1038/s41467-025-65274-1)
Supplement: Supplementary file 2 — Description of Additional Supplementary Files [file 41467_2025_65274_MOESM2_ESM.pdf]

Description of Additional Supplementary Files

Supplementary Data 1: Significant and high confidence chimeras in Hfq RIL-seq analysis

Supplementary Data 2: Differentially expressed genes in response to SisA or CrfA overexpression in ΔvanAB or ΔvanAB ΔsisA-D
