# Supplementary material for: An RNA sponge directs the transition from feast to famine in Caulobacter crescentus
Source: Nat Commun. 2025 Oct 27;16:9478. doi: 10.1038/s41467-025-65274-1 (PMC12559287; doi:10.1038/s41467-025-65274-1)
Supplement: Supplementary file 5 — Reporting Summary [file 41467_2025_65274_MOESM5_ESM.pdf]

nature portfolio

Corresponding author(s): Kathrin S. Fröhlich

Last updated by author(s): Sep 24, 2025

Reporting Summary

Nature Portfolio wishes to improve the reproducibility of the work that we publish. This form provides structure for consistency and transparency in reporting. For further information on Nature Portfolio policies, see our Editorial Policies and the Editorial Policy Checklist.

Statistics

For all statistical analyses, confirm that the following items are present in the figure legend, table legend, main text, or Methods section.

n/a	Confirmed	
☐	☒	The exact sample size (*n*) for each experimental group/condition, given as a discrete number and unit of measurement
☐	☒	A statement on whether measurements were taken from distinct samples or whether the same sample was measured repeatedly
☐	☒	The statistical test(s) used AND whether they are one- or two-sided<br>*Only common tests should be described solely by name; describe more complex techniques in the Methods section.*
☒	☐	A description of all covariates tested
☐	☒	A description of any assumptions or corrections, such as tests of normality and adjustment for multiple comparisons
☐	☒	A full description of the statistical parameters including central tendency (e.g. means) or other basic estimates (e.g. regression coefficient) AND variation (e.g. standard deviation) or associated estimates of uncertainty (e.g. confidence intervals)
☐	☒	For null hypothesis testing, the test statistic (e.g. *F*, *t*, *r*) with confidence intervals, effect sizes, degrees of freedom and *P* value noted<br>*Give P values as exact values whenever suitable.*
☒	☐	For Bayesian analysis, information on the choice of priors and Markov chain Monte Carlo settings
☒	☐	For hierarchical and complex designs, identification of the appropriate level for tests and full reporting of outcomes
☒	☐	Estimates of effect sizes (e.g. Cohen's *d*, Pearson's *r*), indicating how they were calculated

Our web collection on statistics for biologists contains articles on many of the points above.

Software and code

Policy information about availability of computer code

Data collection	Next-generation sequencing data was collected by Illumina NextSeq1000 in single-read mode for RNA-seq transcriptome analysis and paired-read mode for RIL-seq libraries.

Data analysis	Transcriptome RNA-seq computational analysis<br>Read files in FASTQ format were imported into CLC Genomics Workbench (version 22.0.2., Qiagen), trimmed and mapped to the Caulobacter crescentus NA1000 reference genome (NC_011916) using the "RNA-Seq Analysis" tool with standard parameters. Read counts were normalized (CPM) and transformed (log2). Differential expression was tested using the built-in tool corresponding to edgeR in exact mode with tagwise dispersions.<br>RIL-seq computational analysis<br>The de-multiplexed raw reads were analyzed with ChimericFragments (Siemers et al., 2024; https://github.com/maltesie/ChimericFragments) using default parameters except for the minimum seed length and the minimum alignment score (min_seed_len=13, min_alignment_score=17). The resulting table of interactions was filtered according to a minimum read count of 3 and a maximum complementarity FDR of 0.5. The genome sequence and annotation of C. crescentus used in the analysis were downloaded from NCBI RefSeq (NC_011916.1).<br>Band intensities of Western and Northern blots were quantified using the AIDA Image Analyzer Software (version 5.1., raytest) or Fiji / Image J (version 1.53c; https://github.com/imagej/ImageJ/releases/tag/v1.53c).<br>The MultAlin algorithm (version 5.4.1.; http://multalin.toulouse.inra.fr/) was used to align sequences.<br>The RNAfold WebServer (version 2.6.3.; http://rna.tbi.univie.ac.at/cgi-bin/RNAWebSuite/RNAfold.cgi) was employed to predict RNA secondary structures.<br>The IntaRNA algorithm (version 3.4.1.; http://rna.informatik.uni-freiburg.de/IntaRNA/) was used to predict RNA base-pairing interactions.<br>Statistics were calculated using Graphpad Prism (version 10.0.2., Graphpad software).

For manuscripts utilizing custom algorithms or software that are central to the research but not yet described in published literature, software must be made available to editors and reviewers. We strongly encourage code deposition in a community repository (e.g. GitHub). See the Nature Portfolio guidelines for submitting code & software for further information.

Data

Policy information about availability of data

All manuscripts must include a data availability statement. This statement should provide the following information, where applicable:

- Accession codes, unique identifiers, or web links for publicly available datasets
- A description of any restrictions on data availability
- For clinical datasets or third party data, please ensure that the statement adheres to our policy

The demultiplexes sequencing data of the RIL-seq experiments have been deposited in the GEO database under accession number GSE283644. The transcriptome analysis by RNA-seq data from this study can be found under the GEO accession code GSE275909. Further raw and analyzed data in support of the results of this study are available upon request. Source data are provided with this paper.

Research involving human participants, their data, or biological material

Policy information about studies with human participants or human data. See also policy information about sex, gender (identity/presentation), and sexual orientation and race, ethnicity and racism.

Reporting on sex and gender	n/a
Reporting on race, ethnicity, or other socially relevant groupings	n/a
Population characteristics	n/a
Recruitment	n/a
Ethics oversight	n/a

Note that full information on the approval of the study protocol must also be provided in the manuscript.

Field-specific reporting

Please select the one below that is the best fit for your research. If you are not sure, read the appropriate sections before making your selection.

☒ Life sciences ☐ Behavioural & social sciences ☐ Ecological, evolutionary & environmental sciences

For a reference copy of the document with all sections, see nature.com/documents/nr-reporting-summary-flat.pdf

Life sciences study design

All studies must disclose on these points even when the disclosure is negative.

Sample size	Sample sizes were determined by field standards, technical constraints and statistical validation. Unless stated otherwise, three biological replicates were chosen to allow statistical analysis with sufficient power and to account for biological variability.
Data exclusions	No data were excluded.

Replication	Experiments were conducted in biological replicates, specified for each case in the manuscript. Western and Northern blots were performed in 3 replicates (unless stated otherwise) and representative blots are shown. Each experiment was performed at least twice independently, excluding the RIL-seq and transcriptome analysis as is standard.
Randomization	Not applicable. Randomization is not standard for the experiments performed.
Blinding	Not applicable. Blinding is not standard for the experiments performed.

Reporting for specific materials, systems and methods

We require information from authors about some types of materials, experimental systems and methods used in many studies. Here, indicate whether each material, system or method listed is relevant to your study. If you are not sure if a list item applies to your research, read the appropriate section before selecting a response.

Materials & experimental systems

n/a	Involved in the study	
☐	☒	Antibodies
☒	☐	Eukaryotic cell lines
☒	☐	Palaeontology and archaeology
☒	☐	Animals and other organisms
☒	☐	Clinical data
☒	☐	Dual use research of concern
☒	☐	Plants

Methods

n/a	Involved in the study	
☒	☐	ChIP-seq
☒	☐	Flow cytometry
☒	☐	MRI-based neuroimaging

Antibodies

Antibodies used	- anti-GFP antibody (Roche #11814460001), 1:1,000 dilution in TBS-T + 3% BSA<br>- anti-FLAG antibody (Sigma #F1804), 1:1,000 dilution in TBS-T + 3% BSA<br>- anti-GroEL antibody (Sigma #G6532), 1:10,000 dilution in TBS-T + 3% BSA<br>- anti-RNA polymerase antibody (1:5,000; mouse; BioLegend #663205), 1:5,000 dilution in TBS-T + 3% BSA
Validation	Information about the anti-GFP antibody: https://www.sigmaaldrich.com/DE/de/product/roche/11814460001<br>Information about the anti-FLAG antibody can be found on the manufacturer's website: https://www.sigmaaldrich.com/DE/de/product/sigma/f1804<br>Information about the anti-GroEL antibody can be found on the manufacturer's website: https://www.sigmaaldrich.com/DE/de/product/sigma/g6532<br>Information about the anti-RNA polymerase antibody can be found on the manufacturer's website: https://www.biolegend.com/en-us/products/direct-blot-hrp-anti-eme-coli-em-rna-sigma-70-antibody-13486

Plants

Seed stocks	n/a
Novel plant genotypes	n/a
Authentication	n/a
