# Supplementary material for: An RNA sponge directs the transition from feast to famine in Caulobacter crescentus
Source: Nat Commun. 2025 Oct 27;16:9478. doi: 10.1038/s41467-025-65274-1 (PMC12559287; doi:10.1038/s41467-025-65274-1)

Source data for Fig. 2e

Northern blot

SisA

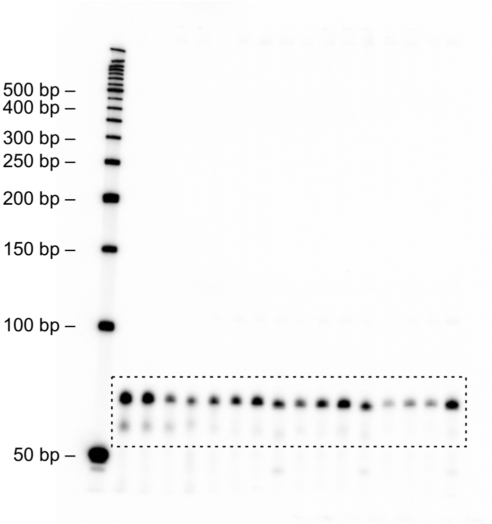

SisB

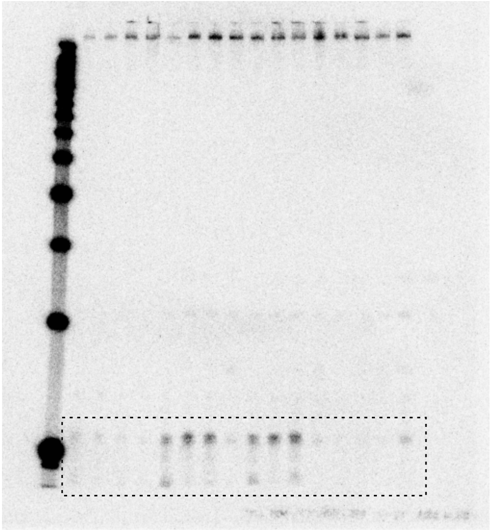

SisC

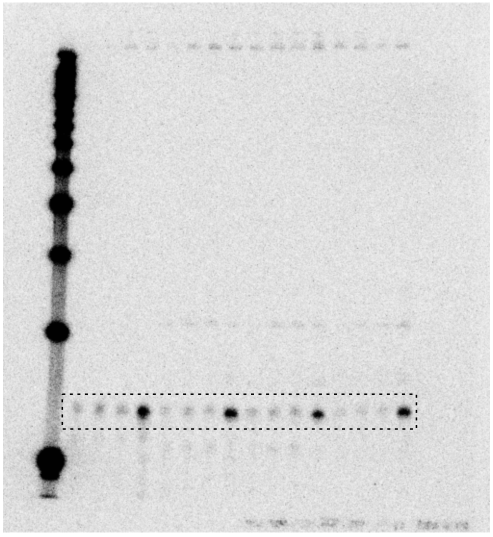

SisD

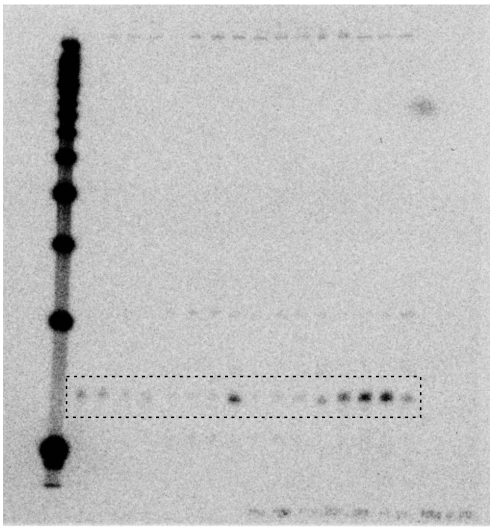

CrfA

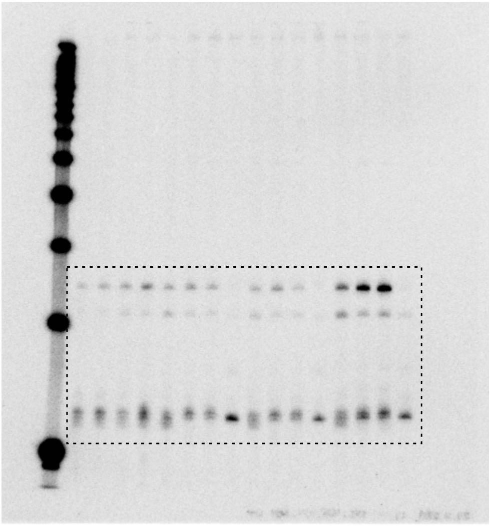

tmRNA

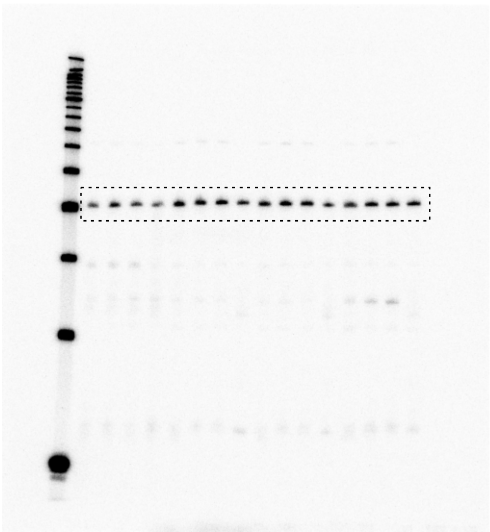

Source data for Fig. 2f

Sequencing gel for CrfA structure probing

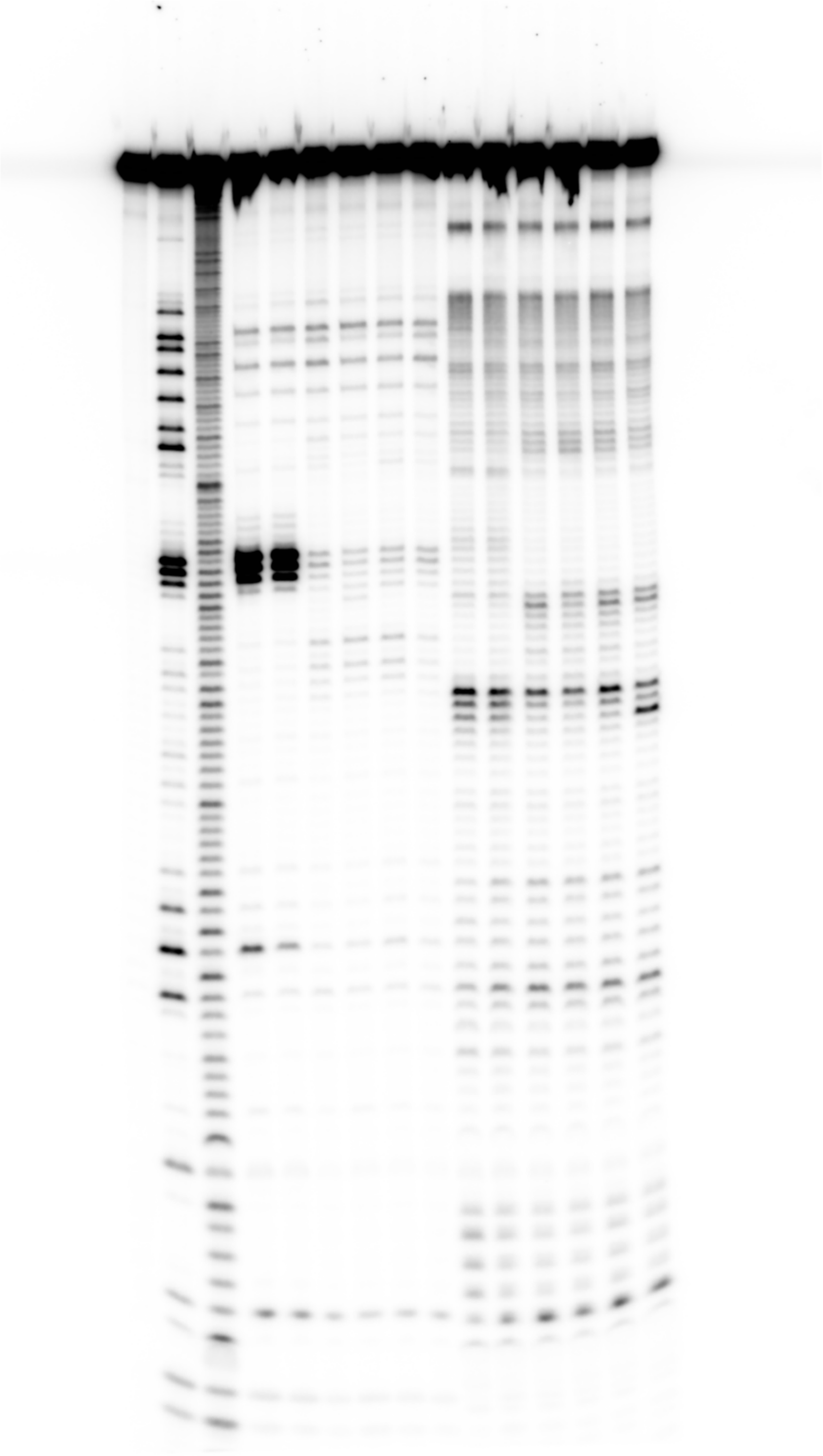

Source data for Fig. 3a

Northern blot

SisA

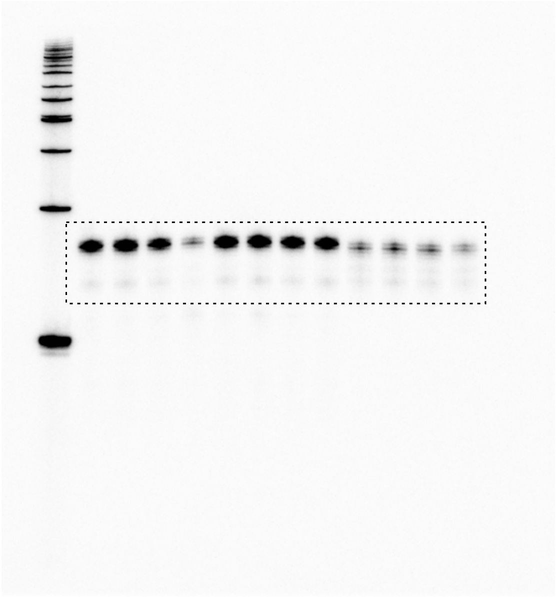

CrfA

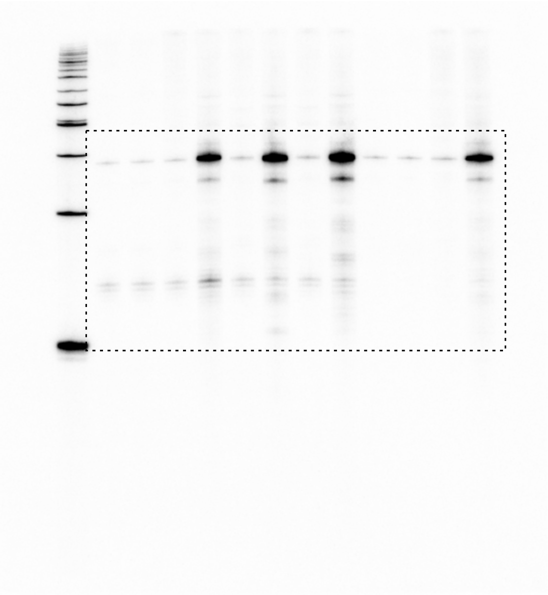

5S

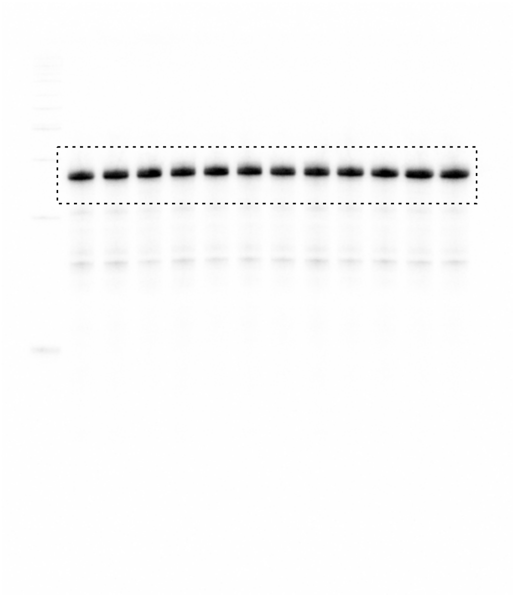

Source data for Fig. 3c

Northern blot

SisA

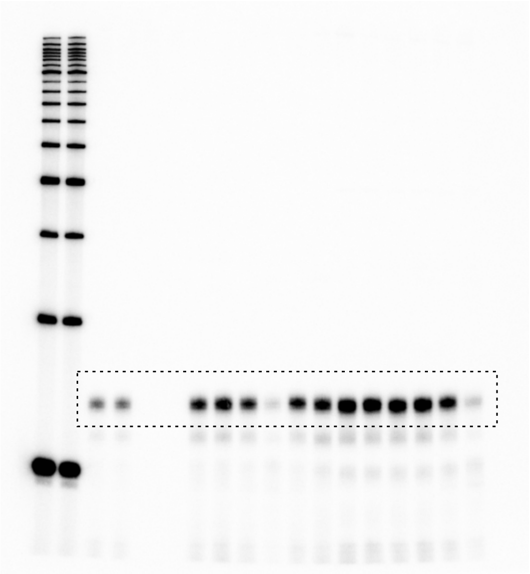

CrfA

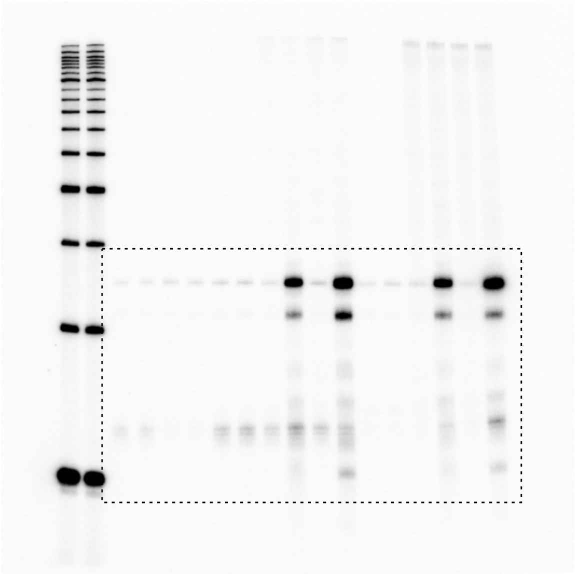

tmRNA

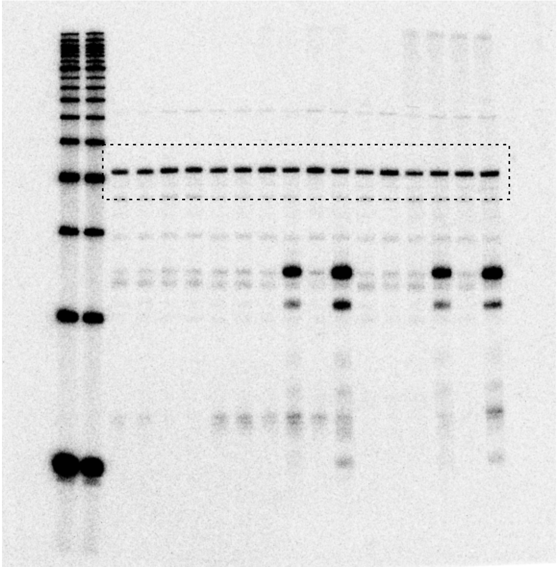

Source data for Fig. 6b

Northern blot

CrfA

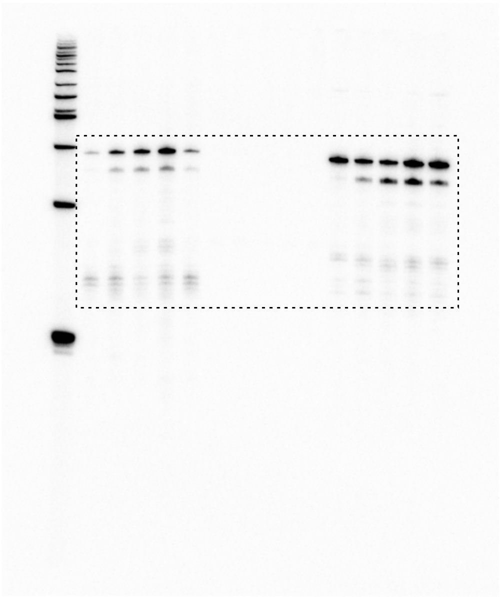

SisA

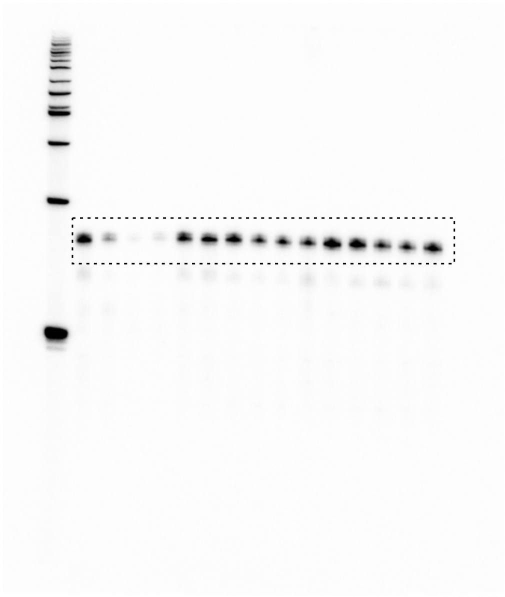

5S

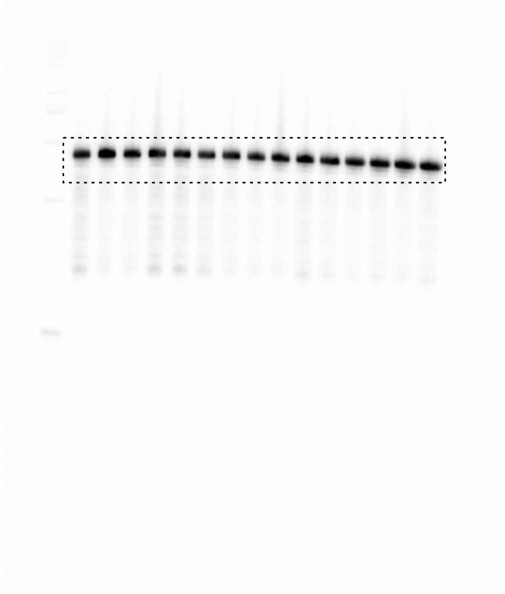

Source data for Fig. 6d

Western blot - CCNA_03574::3xFLAG

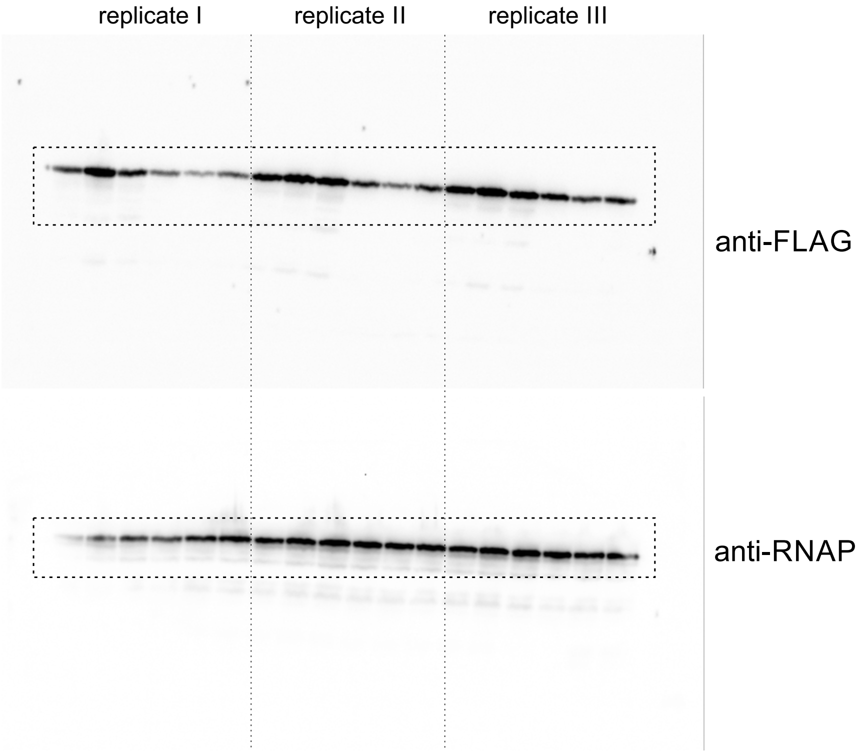

Western blot - CCNA_03263::3xFLAG

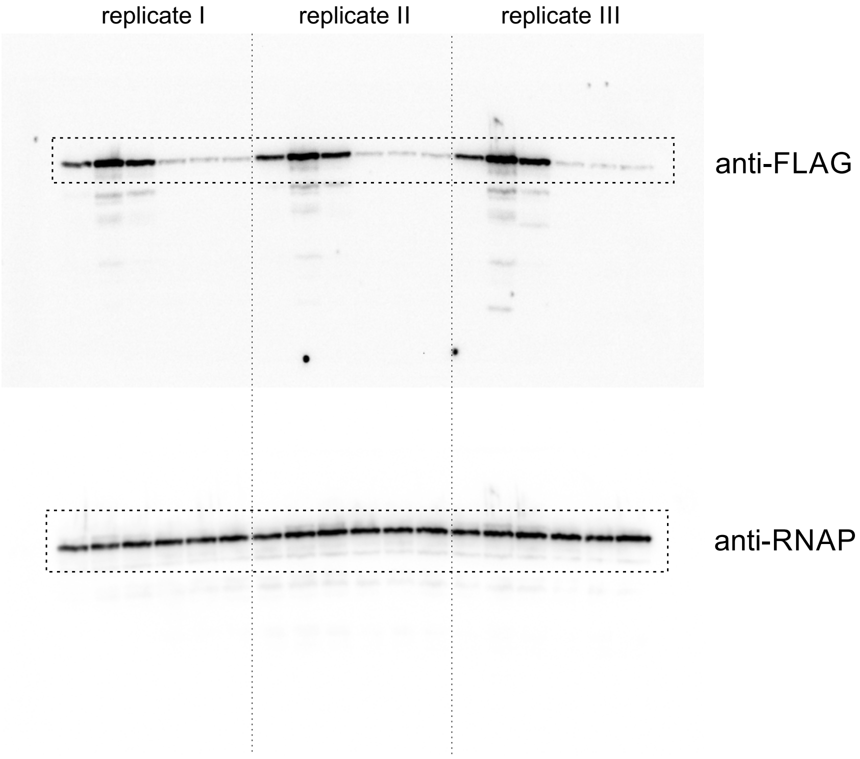

Source data for Fig. 6d

Western blot - CCNA_03444::3xFLAG

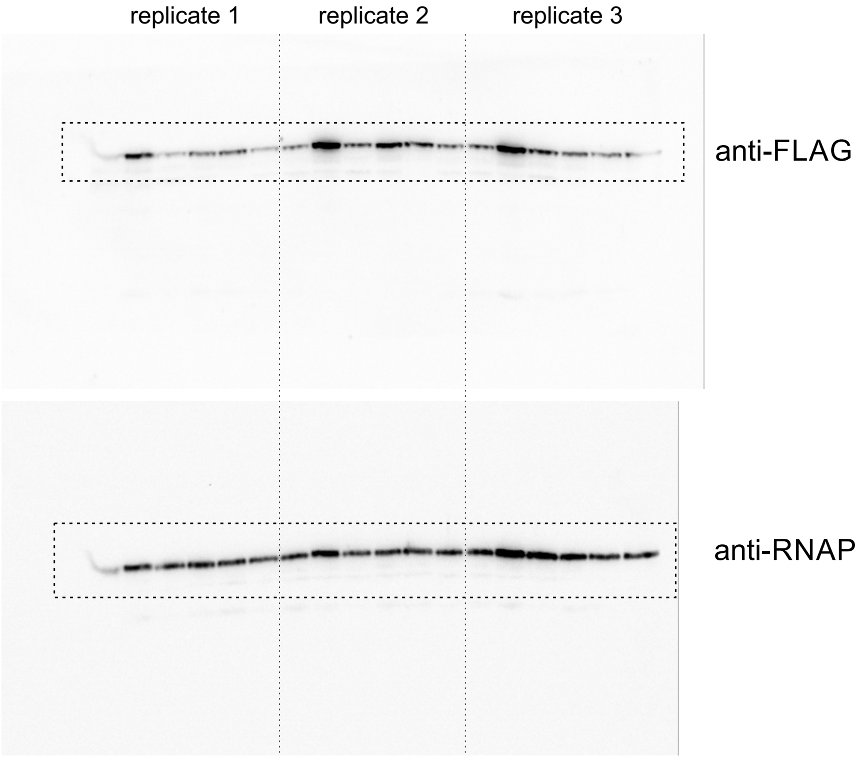

Source data for Fig. S2a

Northern blot

SisA

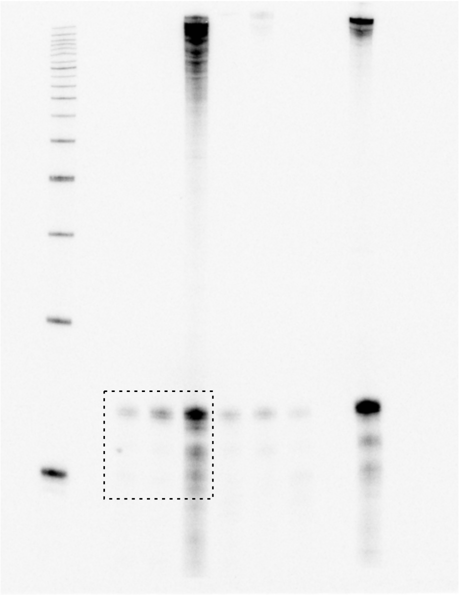

5S

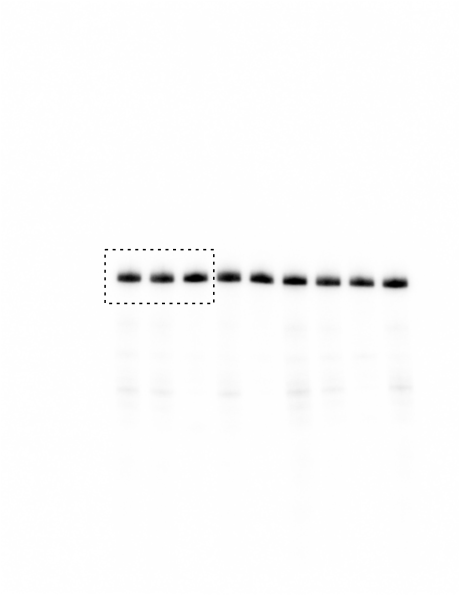

Source data for Fig. S2b

Western blot - CCNA_00338::gfp

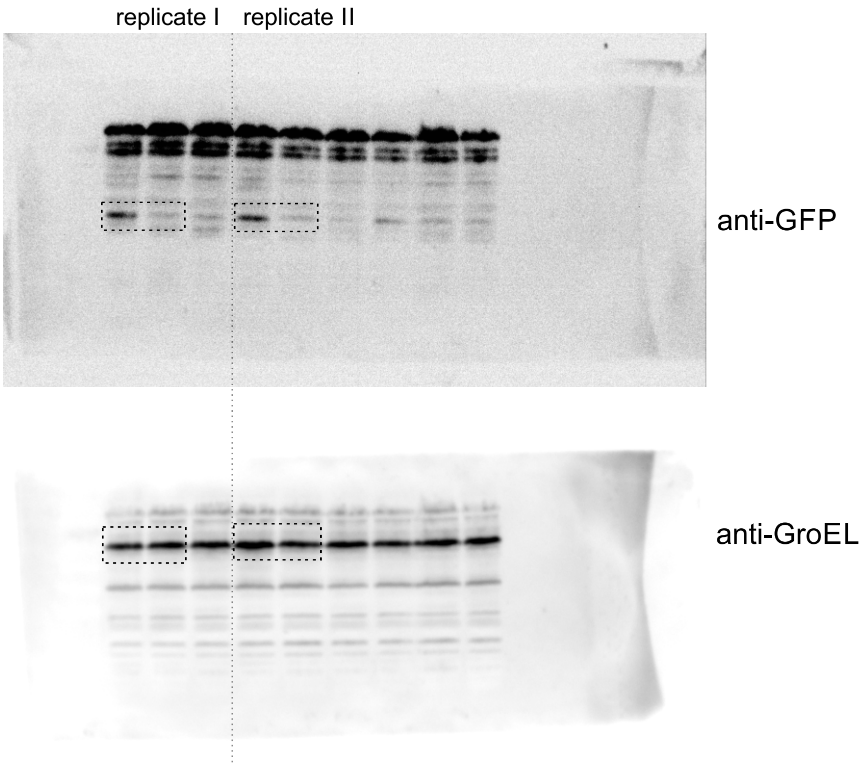

Western blot - CCNA_03263::gfp

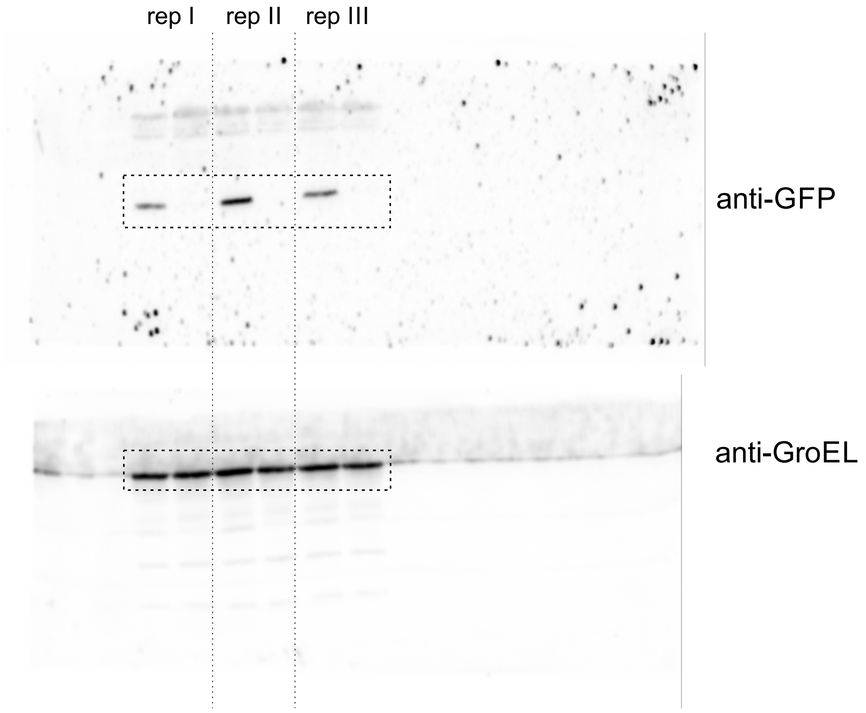

Source data for Fig. S2b

Western blot - CCNA_03444::gfp

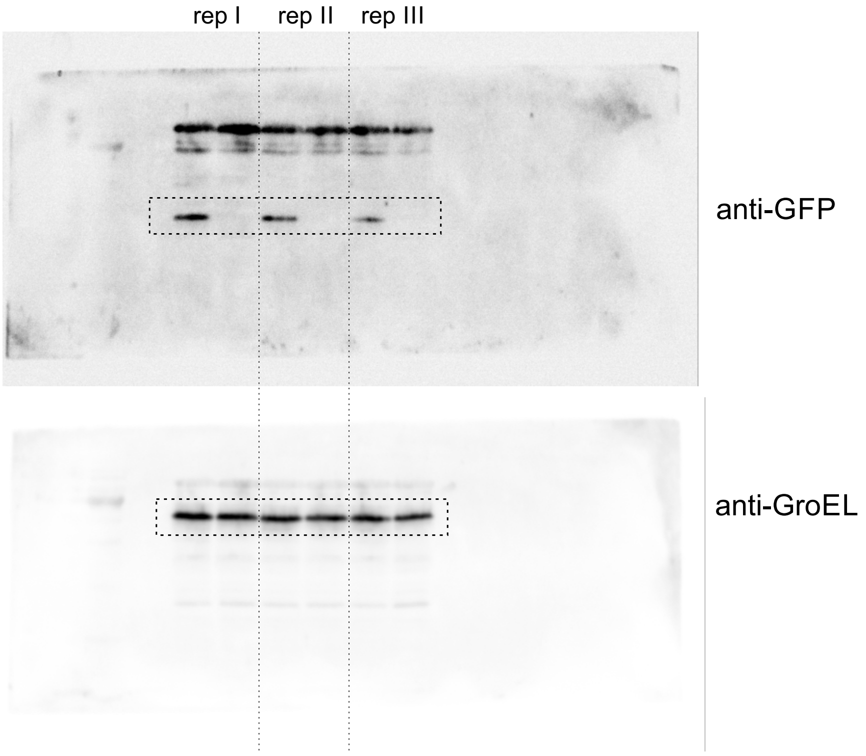

Western blot - gfp

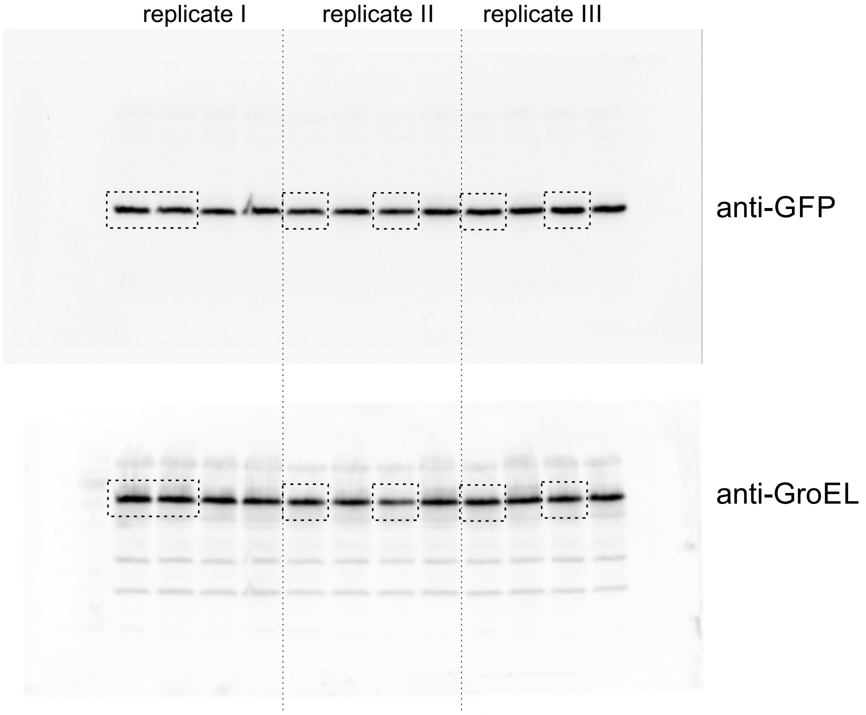

Source data for Fig. S4a

Northern blot with T7 RNA for quantitation

SisA

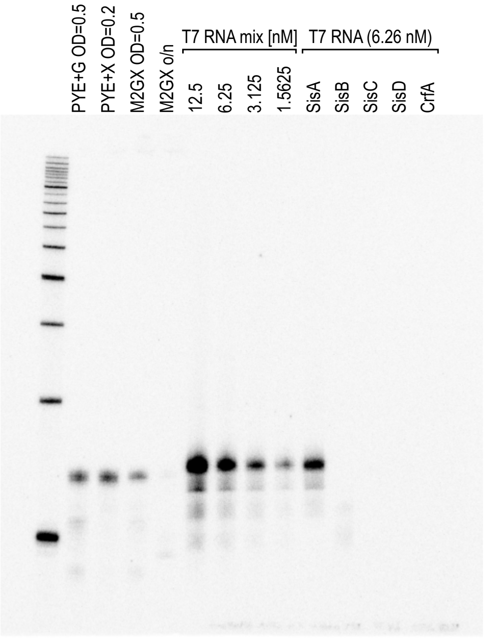

SisB

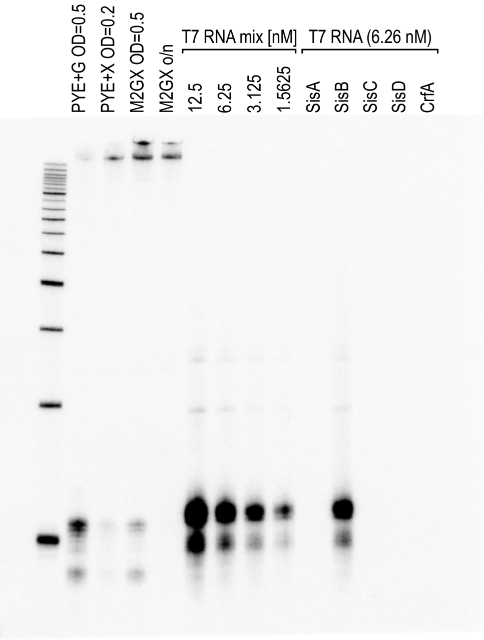

SisC

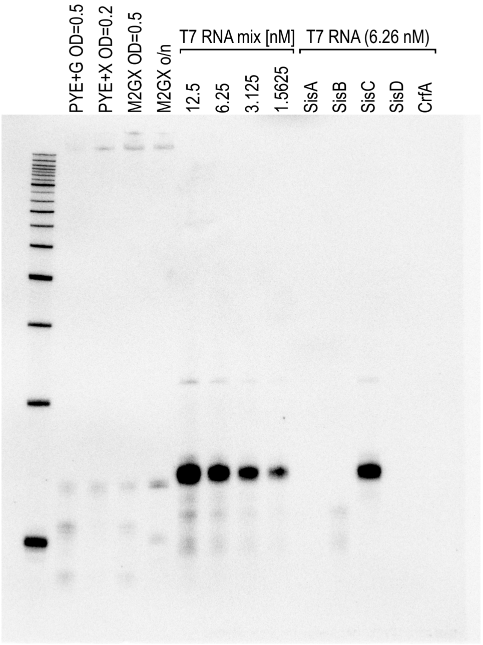

SisD

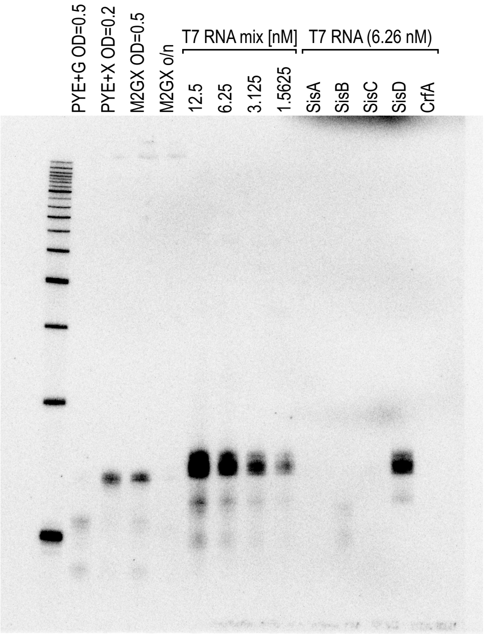

CrfA

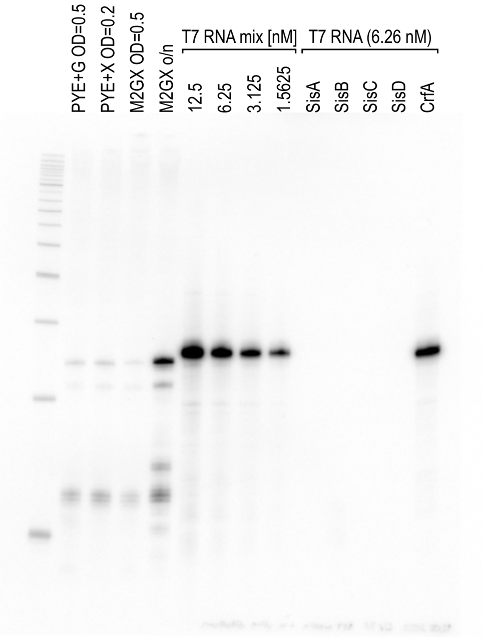

Source data for Fig. S4b

Northern blot

SisA

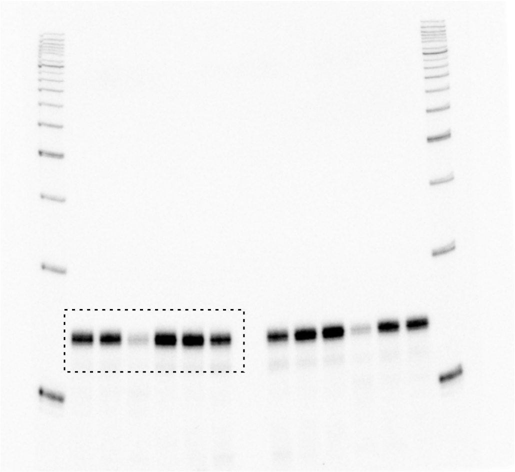

SisB + SisD

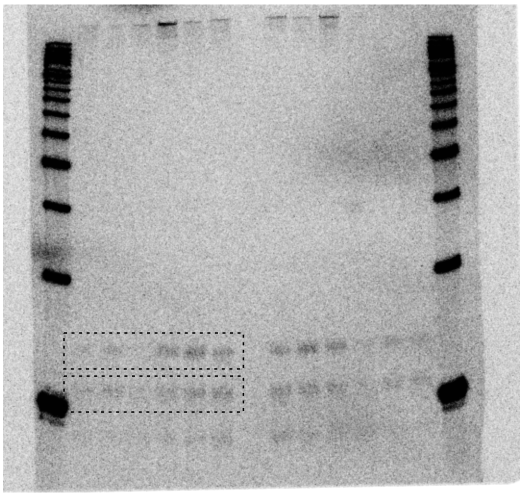

SisC

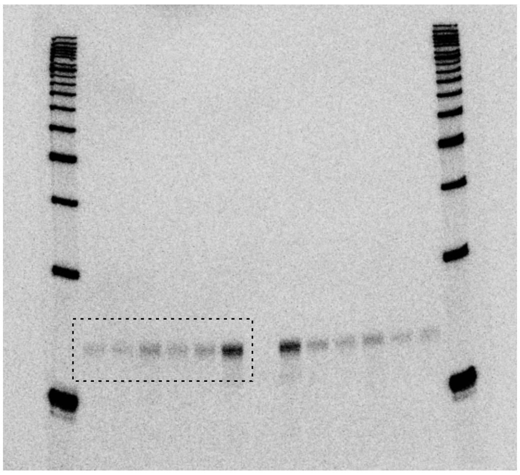

CrfA

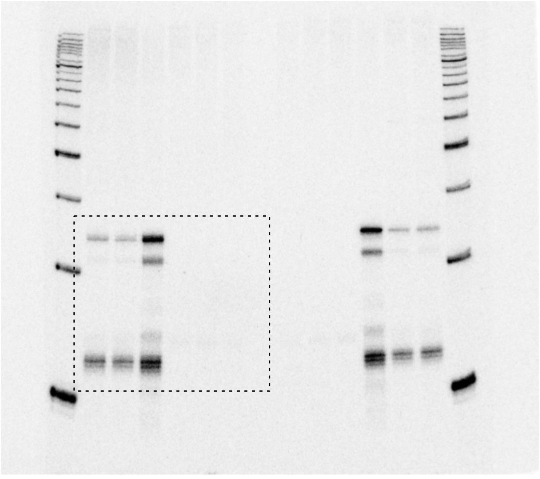

tmRNA

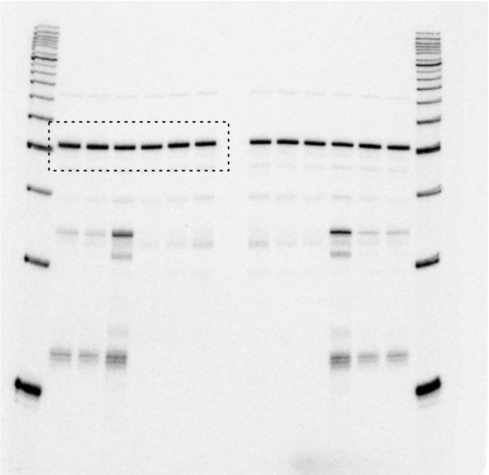

Source data for Fig. S6a

Sequencing gel for SisA structure probing

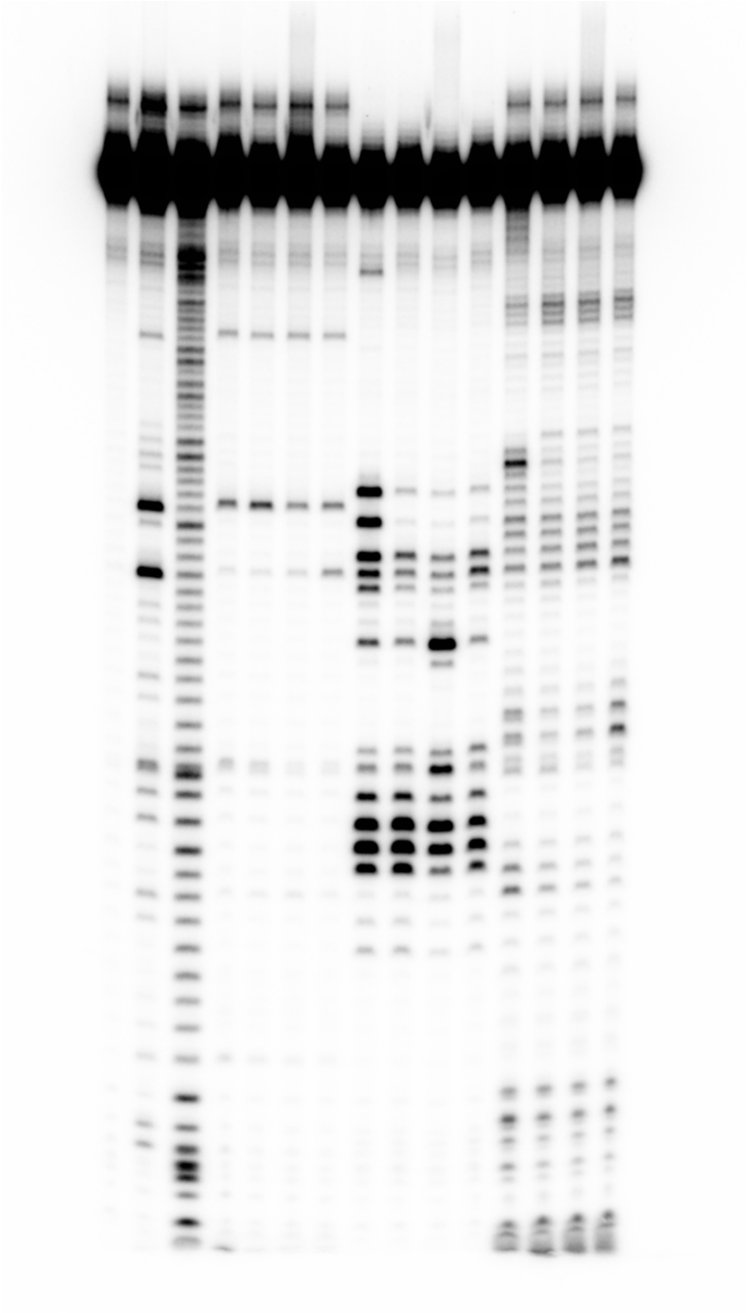

Source data for Fig. S6d

Gel for EMSA of SisA* with Hfq and cold CrfA variants

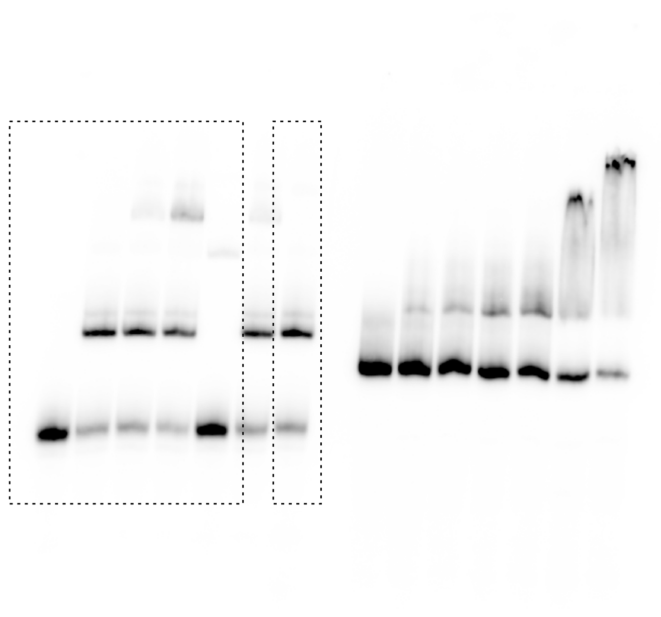

Source data for Fig. S7a

Northern blot

SisA

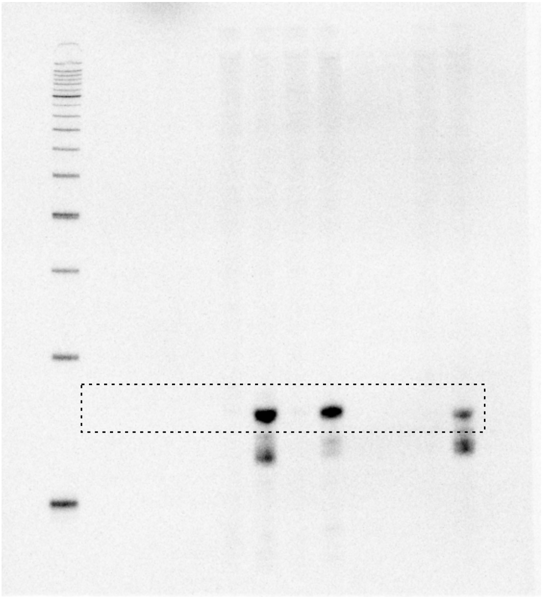

CrfA

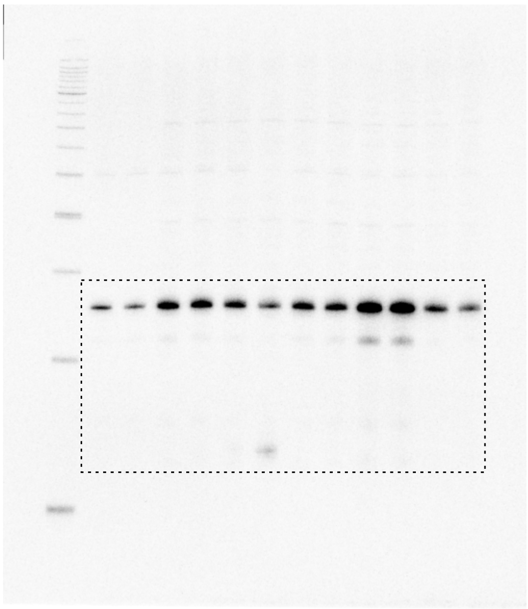

tmRNA

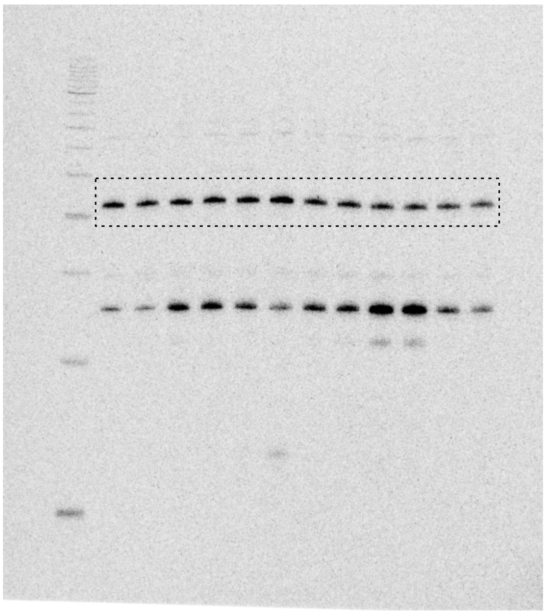

Source data for Fig. S8a

Northern blot

SisA

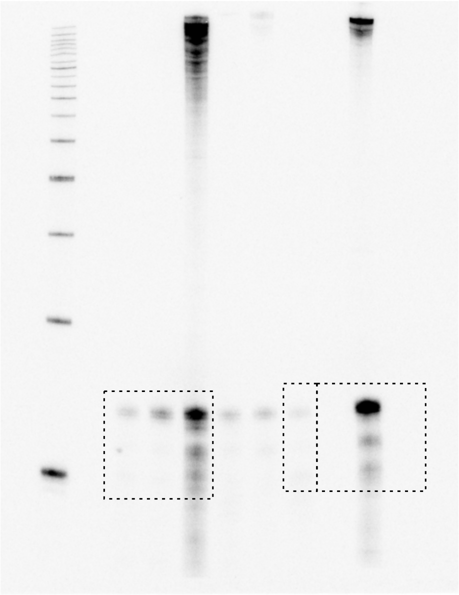

CrfA

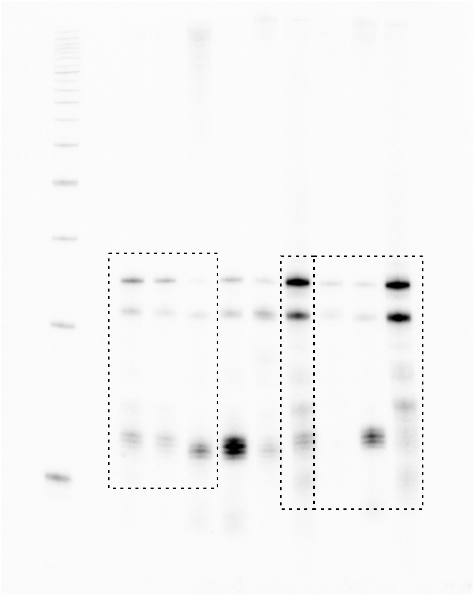

5S

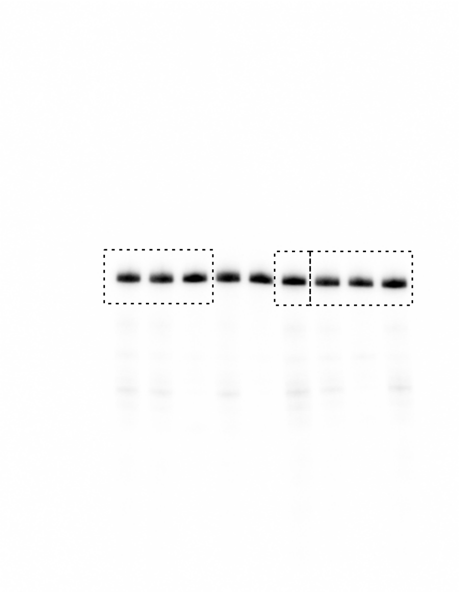

Source data for Fig. S8b

Northern blot

SisA

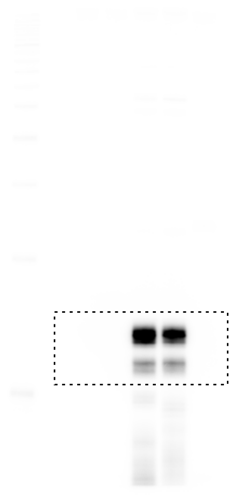

CrfA

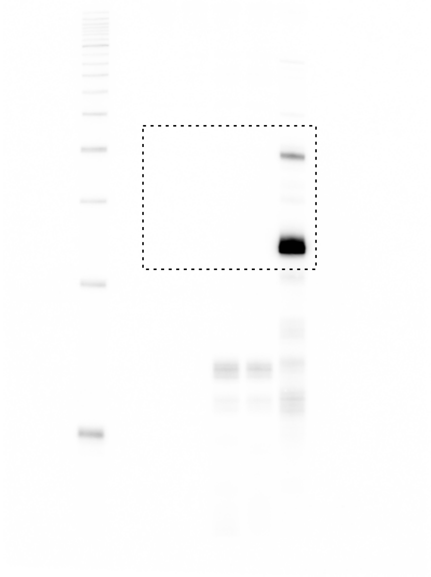

5S

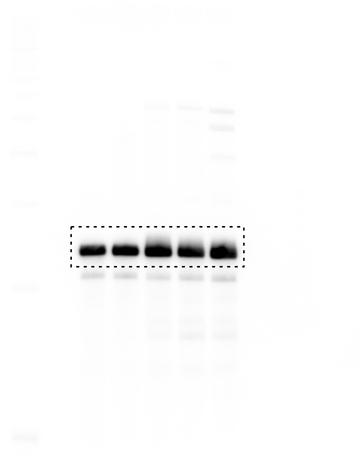

Source data for Fig. S9

Northern blot

SisA

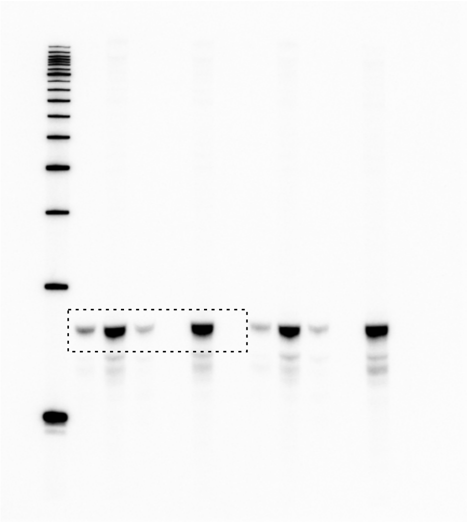

CrfA

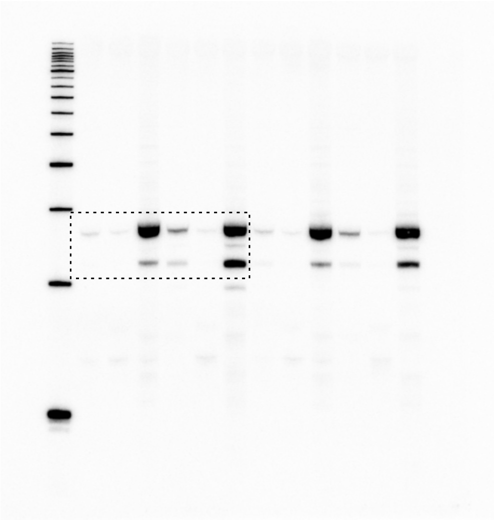

tmRNA

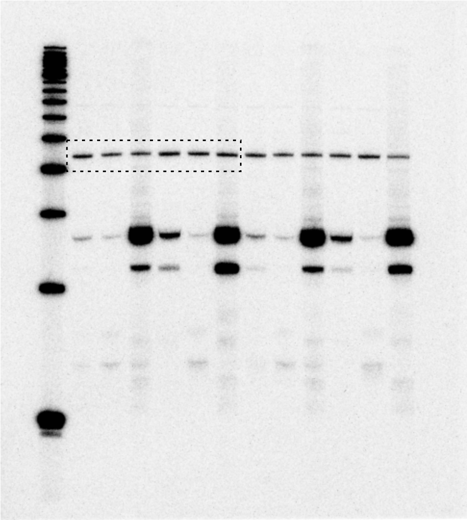

Source data for Fig. S10b

Western blot - CCNA_03444::gfp

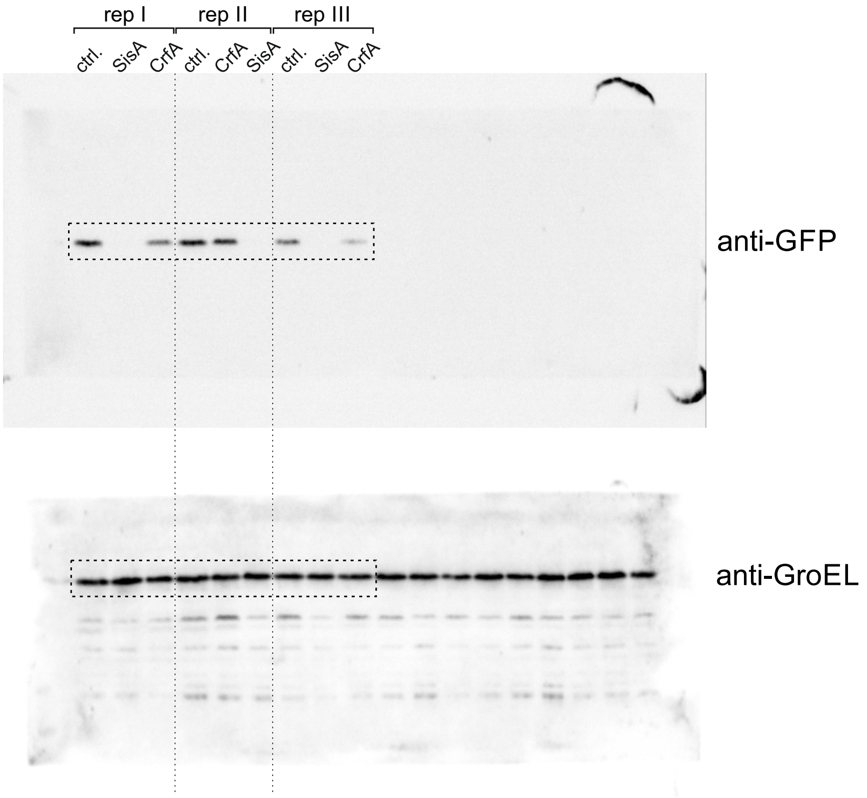

Western blot - CCNA_00338::gfp

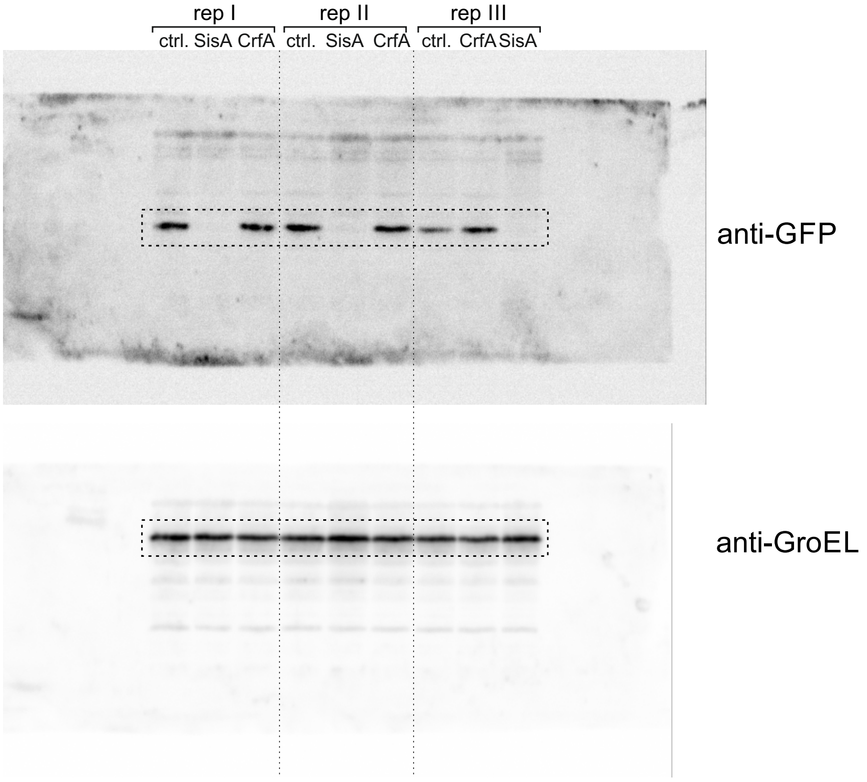

Source data for Fig. S10b

Western blot - gfp

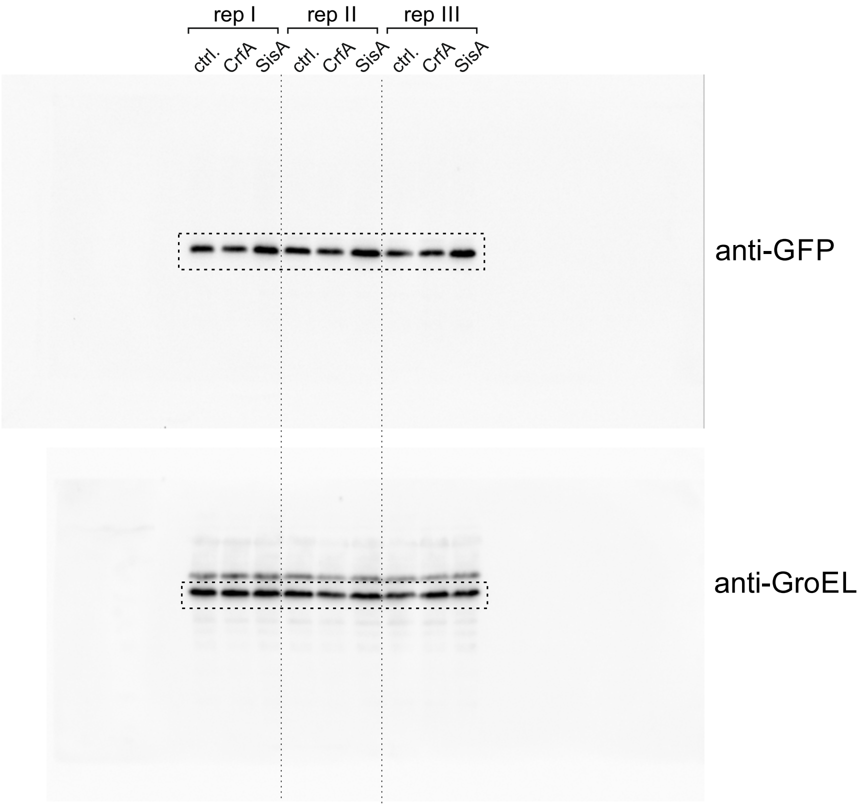

Source data for Fig. S11b

Western blot - HppA::3xFLAG

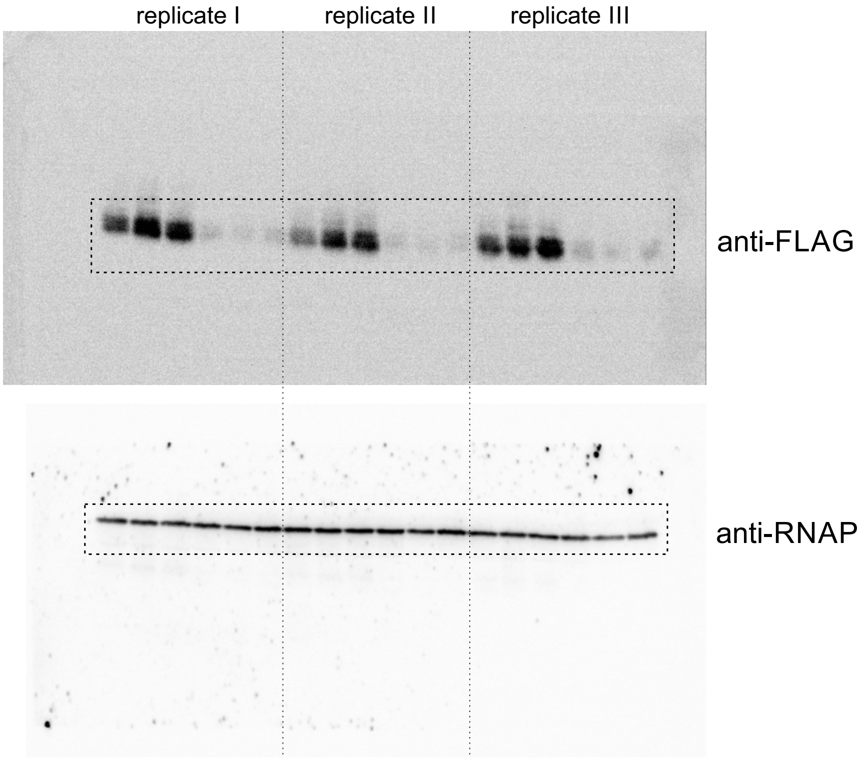

Western blot - CCNA_00338::3xFLAG

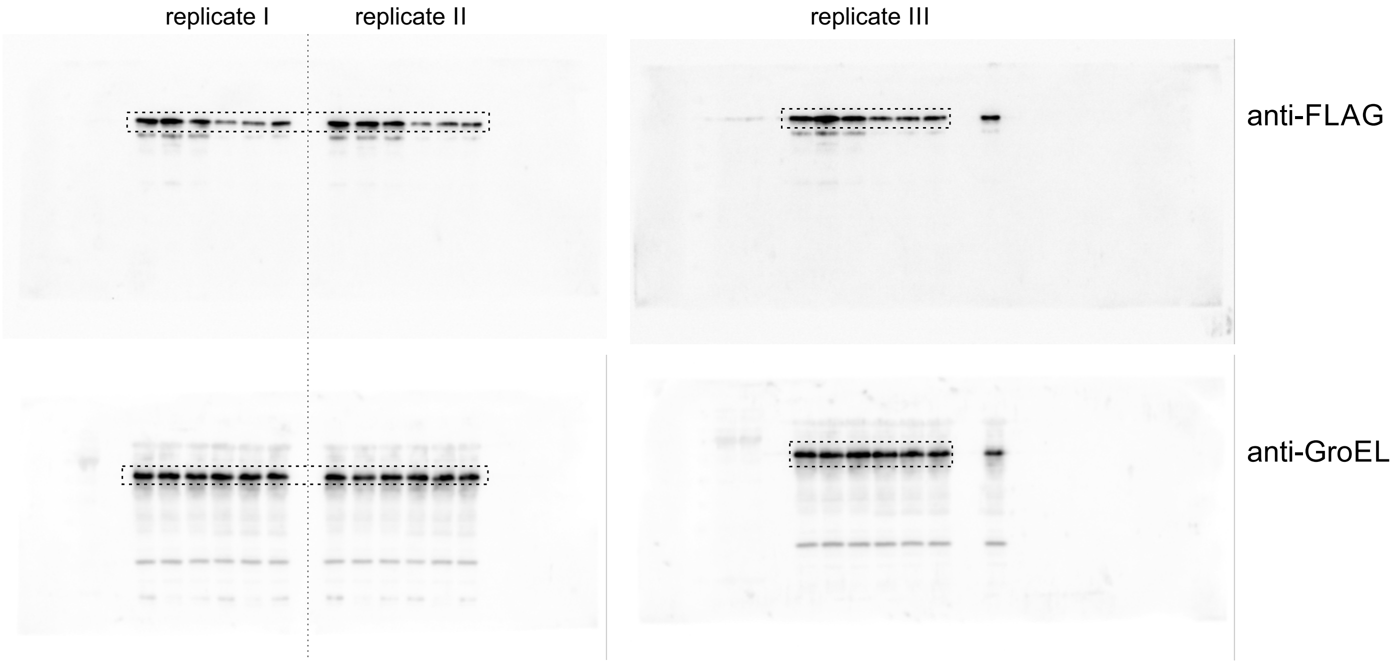

Supplement: Supplementary file 7 — Source data [file 41467_2025_65274_MOESM7_ESM.zip › Source-Data-blots.pdf]
